# Multi-mode movement decisions across widely ranging behavioral processes

**DOI:** 10.1371/journal.pone.0272538

**Published:** 2022-08-11

**Authors:** Marie-Caroline Prima, Thierry Duchesne, Jerod A. Merkle, Simon Chamaillé-Jammes, Daniel Fortin

**Affiliations:** 1 Department of Biology, Université Laval, Québec, QC, Canada; 2 Department of Mathematics and Statistics, Université Laval, Québec, QC, Canada; 3 Wyoming Cooperative Fish and Wildlife Research Unit, Department of Zoology and Physiology, University of Wyoming, Laramie, Wyoming, United States of America; 4 CEFE, Univ Montpellier, CNRS, EPHE, IRD, Montpellier, France; 5 Mammal Research Institute, Department of Zoology & Entomology, University of Pretoria, Pretoria, South Africa; 6 LTSER France, Zone Atelier “Hwange”, Hwange National Park, Dete, Zimbabwe; Texas State University, UNITED STATES

## Abstract

Movement of organisms plays a fundamental role in the evolution and diversity of life. Animals typically move at an irregular pace over time and space, alternating among movement states. Understanding movement decisions and developing mechanistic models of animal distribution dynamics can thus be contingent to adequate discrimination of behavioral phases. Existing methods to disentangle movement states typically require a follow-up analysis to identify state-dependent drivers of animal movement, which overlooks statistical uncertainty that comes with the state delineation process. Here, we developed population-level, multi-state step selection functions (HMM-SSF) that can identify simultaneously the different behavioral bouts and the specific underlying behavior-habitat relationship. Using simulated data and relocation data from mule deer (*Odocoileus hemionus*), plains bison (*Bison bison bison*) and plains zebra (*Equus quagga*), we illustrated the HMM-SSF robustness, versatility, and predictive ability for animals involved in distinct behavioral processes: foraging, migrating and avoiding a nearby predator. Individuals displayed different habitat selection pattern during the encamped and the travelling phase. Some landscape attributes switched from being selected to avoided, depending on the movement phase. We further showed that HMM-SSF can detect multi-modes of movement triggered by predators, with prey switching to the travelling phase when predators are in close vicinity. HMM-SSFs thus can be used to gain a mechanistic understanding of how animals use their environment in relation to the complex interplay between their needs to move, their knowledge of the environment and navigation capacity, their motion capacity and the external factors related to landscape heterogeneity.

## Introduction

Movement decisions of animals are driven by a complex interplay between their need to move (e.g., food, shelter), the knowledge of the environment and navigation capacity (e.g., move towards a known-rich area), the motion capacity (e.g., distance travelled per time unit) and the external factors related to landscape heterogeneity (e.g., movement barriers, corridors) [1, 2]. Using a relevant spatio-temporal scale, different phases of movement can be extracted from trajectories of geolocalised individuals, each reflecting a behavioral state that can influence the outcome of this complex interplay [3, 4]. For example, when foragers encounter a resource patch, movement can become tortuous and slow, reflecting an area-restricted search [5], whereas movement among resource patches are fast and relatively straight, depicting the crossing of unsuitable foraging conditions [6]. These two movement modes have been called “encamped” and “exploratory” or “travelling” [7, 8]. Therefore, multi-state analysis applied to animal trajectories can be used to infer foraging phases through time, and provide information on the spatio-temporal scale over which this foraging process occurs [3, 9].

Foraging and exploratory movements are not the only behaviors that can be identified from multi-state analysis applied to individual trajectories. For example, Cagnacci, Focardi [10] used characteristics of animal trajectories from five deer species to identify migratory and non-migratory individuals together with the timing of migration, distance travelled and time spent in the summer range. Such information is useful to understand space use strategies allowing animals to exploit their temporally variable and spatially heterogeneous environments [11]. Since our understanding of the movement process might be biased when biologically relevant behaviors are omitted [12], the inclusion of more detailed and realistic behaviors into movement models is also important to better understand how animals perceive and react to their environment. For example, African wild dogs (*Lycaon pictus*) selected roads during their travelling modes but avoided them during resting periods [7]. This behavior-specific response to roads was, however, not detected when behavioral states were not considered in the selection analysis [7].

Various mathematical and statistical tools have been used to identify behavioral bouts from movement data, such as mixtures of random walks [6, 13], first passage time [3], semivariance function [14], hidden Markov model [4], state-space model [15], k-means clustering coupled with gap statistic [16], and acceleration tri-axial coupled with machine learning [17]. A criticism of several of these approaches (e.g., first passage time, behavioral change point analysis, 3, 11) is their lack of inclusion of spatial information, such as habitat attributes, to identify behavioral bouts, an omission that limits our understanding of behavioral states in a biological meaningful way [18, 19]. Mixtures of random walks or state space models can include landscape features to assess how habitat attributes influence switching rates [13], and attract or repulse animal depending on the movement mode [20, 21]. However, the implementation and computational challenges required with such models often limit the number of parameters that could be estimated [8, 19, 22]. Besides, such mechanistic models must assume an underlying movement process [e.g., biased or correlated random walk, 15, 21], which can induce a discrepancy between real and inferred animal movement behavior in case of misspecification [18, 22].

An alternative way to assess the behavior-specific response to habitat attributes consists of performing habitat selection analysis as a second step [23, 24]. Such a 2-step analysis approach first identified encamped and exploratory modes of movement in elephants (*Loxodonta Africana*) using a multi-state random walk movement model, followed by resource selection functions applied to each state, to illustrate behavior-dependent habitat selection by elephants [25]. Although this approach was a first step towards a better mechanistic understanding of the selection process, behavioral states were identified using only the characteristics of the movement path (i.e., movement rate and turning angle) but no habitat covariates [7, 23, 25], thereby risking incorrect inferences. Indeed, movement modes were assumed to be accurately known when proceeding to the resource selection analysis whereas in fact, they were estimated from a multi-state random walk movement model such that movement modes were uncertain. This uncertainty was, however, not considered in the resource selection functions such that the error associated with the selection coefficient estimates might have been underestimated when proceeding in a 2-step analysis.

Here we develop population-level models that account for both animal movement and local habitat selection in a multi-state setting. The approach combines step selection functions [26] and hidden Markov models to generate multi-state SSF (HMM-SSF) yielding state-specific movement-habitat relationships [27]. A substantial weakness of the current HMM-SSF methodology is that it provides guideline only to model the movements a single individual, which decreases its value for ecological research by preventing robust inference at the population level. Here we show how to combine the HMM-SSF from several individuals to infer habitat selection at the population level. Further, we demonstrate how to fit trajectories of individuals based on irregular time intervals while including missing data, and how to test the predictive capacity of the model using *k*-fold cross validation calculated for each behavioral state. We then illustrate the versatility of HMM-SSF’s applicability to multiple ecological systems using simulated and empirical movement datasets. We simulate three ecological scenarios from state-specific step selection functions and three ecological scenarios from multi-state biased correlated random walks to assess the ability and robustness of the HMM-SSF at detecting different movement modes and state-specific habitat selection under different assumptions. We also compare state-specific habitat selection estimates from the HMM-SSF and the 2-step approach. We then apply the HMM-SSF to empirical trajectories to: 1) identify the onset of mule deer (*Odocoileus hemionus*) migration together with habitat selection during the different phases of the migratory behavior; 2) evaluate behavior-habitat relationships during movement modes of two forager species (plains bison, *Bison bison bison* and zebra, *Equus quagga*); 3) assess the circumstances over which a forager (plains bison) flees from a predator (wolf, *Canis lupus*) by identifying environmental factors influencing transitions from encamped to travelling mode of movement.

## Materials and methods

### HMM-SSF overview

We suppose that we obtained *T* steps from *T*+1 geolocations collected every hour from one individual. We define *Z*_*t*_ = (*x*_*t*_, *x*_*t*+1_; *y*_*t*_, *y*_*t*+1_), *t ϵ* {1,…,*T*}, the observed step at time *t*. In a single-state step selection function analysis (SSF), a set of *J* random stepsare drawn for each observed step *Z*_*t*_. Characteristics of the landscape and the movement path (i.e., turning angle and step length), thereafter called covariates, can be compared between observed and random steps in a discrete choice model framework [26], allowing to mechanistically assess how animal uses its habitat while moving. We define ***X***_***jt***_, the vector with the value of *q* covariates for the *j*-th (observed or random) step at time *t*, *j* = 0, …, *J*, with index *j* = 0 denoting the observed step.

In HMM-SSF, we consider that an individual can be in *K* different movement modes (i.e., behavioral phases or states) varying through time, each exhibiting a single-state step selection behavior. Let *S*_*t*_ be the unobserved movement mode at step *t*. We assume that *S*_*t*_ is a *K*-state hidden Markov chain with transition probability matrix *π*. The (*i*, *j*)-th element of *π* is *γ*_*ij*_ = ℙ(*S*_*t*+1_ = *j*|*S*_*t*_ = *i*), *i*, *j ε* {1,…,*K*}^2^. The state-specific conditional likelihood of the observations is given by

$$P(Z_{t}|S_{t}=k)=\frac{exp(X_{0t}^{T}\beta^{k})}{\sum_{j=0}^{J}exp(X_{jt}^{T}\beta^{k})},$$ (1)

where ***β***^***k***^, *k ϵ* {1,…,*K*}, is the vector of *q* selection coefficients of the SSF when the animal is in mode *k*. We use $P(S_{t}=k|F_{t-1}^{0})$ to denote the conditional probability to be in state *k* at step *t* given the observed data history up to step $t-1,F_{t-1}^{0}$. The interpretation of model parameter ***β***^***k***^ are similar to the ones of a single-state SSF, except that they are now state-specific [26, 27].

The model parameter *γ*_*ij*_ indicates the constant probability of switching from movement mode *i* to movement mode *j* between step *t* and step *t*+1. If *γ*_*ij*_ is close to 1 and the animal is in movement mode *i* at one step, it then has high odds to switch to movement mode *j* the next step. If $P(S_{t}=k|F_{t-1}^{0})$ is close to 1, the animal is likely moving in mode *k* at step *t*, whereas if $P(S_{t}=k|F_{t-1}^{0})$ is close to 0, the animal is likely moving in a different mode at step *t*.

### Dealing with irregular time intervals and missing data

We now suppose that we obtained geolocations of one individual every three hours two days a week, and every hour five days a week. As model parameters are defined and interpretable for a particular time interval, we cannot adjust the model using the dataset including both the 3-hour observations and the 1-hour observations. A solution would be to resample the 1-hour observations to every three hours to obtain at the end only one-time interval. However, this approach creates a loss in accuracy of the individual’s trajectory and missing data could still remain after the resampling. Another solution would be to only keep the 1-hour observations. In such case, groups of data at 1-hour intervals are each separated from the next one by a gap of two days. Consequently, calculation of $P(S_{t}=k|F_{t-1}^{0})$ is more complex. To approximate the general likelihood of Eq 2.3 in [27] in this condition, we first calculated the likelihood of each group of successive steps, then we multiplied the likelihoods of all groups. We thereby implicitly treated the groups of successive steps as independent. Selection coefficient estimates will not be impacted by a violation of this assumption, although estimates of variance of selection coefficients can be underestimated. An evaluation of the predictive performance of the HMM-SSF (see section **Evaluation of model predictive capability**) should thus be performed to assess the validity of the inferences.

### Population level estimates

We now fit the HMM-SSF separately to *N* trajectories from *N* independent individuals. We thus obtain *N* vectors $\hat{\beta_{n}}=(\hat{\beta_{n}^{1},}\hat{\beta_{n}^{2},}\ldots ,\hat{\beta_{n}^{K}}),n\;\epsilon \;\{1,\ldots ,N\}$ each comprised of *p* =*Kq* selection coefficients estimates, i.e., *q* for each of the *K* modes of movement, and *N* associated variance-covariance matrices $\hat{V_{n}},n\;\epsilon \;\{1,\ldots ,N\}$ of size p*p. We then define $\hat{\beta_{all}}={\left({\hat{\beta_{1}}}^{T},\ldots ,{\hat{\beta_{N}}}^{T}\right)}^{T}$, the vector of selection coefficients estimates from *N* individuals and $\hat{V_{all}}$ the corresponding block diagonal matrix comprised of the *N* individual-level variance-covariance matrices,

$$\hat{V_{all}}=\left[\begin{array}{ccc} \hat{V_{1}} & \cdots & 0\\ \vdots & \ddots & \vdots \\ 0 & \cdots & \hat{V_{N}} \end{array}\right]$$ (2)

We also compute the following matrix:

$$Q=\left(\begin{array}{ccc} 1 & \ldots & 0\\ \vdots & \ddots & \vdots \\ 0 & \cdots & 1\\ & \vdots & \\ 1 & \ldots & 0\\ \vdots & \ddots & \vdots \\ 0 & \cdots & 1 \end{array}\right)=I_{p}⨂1_{N},$$ (3)

the Kronecker product of the identity matrix of size p*p, ***I***_***p***_, and a vector of 1 of length *N*, **1**_***N***_.

Population level coefficients estimates, $\hat{\beta_{Pop}}$, can be estimated from a weighted average of individual coefficients, using the inverse of the variance as weights [28], viz.

$$\hat{\beta_{Pop}}={\left(Q^{T}{\hat{V_{all}}}^{-1}Q\right)}^{-1}Q^{T}{\hat{V_{all}}}^{-1}\hat{\beta_{all}}$$ (4)

and associated variance-covariance matrix, $\hat{V_{Pop}}$, is estimated using:

$$\hat{V_{Pop}}={\left(Q^{T}{\hat{V_{all}}}^{-1}Q\right)}^{-1}.$$ (5)

Using this approach implicitly takes into account the within-animal correlation to estimate population level coefficients estimates [28].

### Evaluation of model predictive capability

Following [29], we suggest to evaluate the predictive capability of the HMM-SSF using *k*-fold cross validation. First, one HMM-SSF can be fitted to each individual’s trajectory, thereby leading to a set of state-specific selection coefficient estimates, $\hat{\beta_{n}^{k}},\;k\;\epsilon \;\{1,\ldots ,K\}$, for each individual *n ϵ* {1,…,*N*}. The conditional probability to be in state *k*, *k ϵ* {1,…,*K*}, at step *t*, *t ϵ* {1,…,*T*}, given the observed data (i.e., $P(S_{t}=k|F_{t-1}^{0}))$ can be used to predict the movement mode at each step *t* of the individual’s trajectory. Because $P(S_{t}=k|F_{t-1}^{0})$ ranges between 0 and 1, a threshold needs to be set to define the mode at each step. The movement mode assigned at each step was the one with the highest probability (i.e., the most probable) given the observed data. For example, in case of two states, if $P(S_{t}=1|F_{t-1}^{0})\geq 0.5$, the individual is assumed to be in state 1 at step *t* and, in state 2 otherwise. Then, one can randomly sample 80% of individuals and use their state-specific selection coefficient estimates to compute state-specific population level estimates, $\hat{\beta_{Pop}^{k},}\;k\;\epsilon \{1,\ldots ,K\}$, using Eq 4. Then, $\hat{\beta_{Pop}^{k},}\;k\;\epsilon \{1,\ldots ,K\}$ can be used to predict state-specific selection probabilities (i.e., ℙ(*Z*_*t*_|*S*_*t*_ = *k*), see Eq 1) along the 20% of non-sampled individual trajectories, for both observed and random steps. By doing so, we evaluate the performance of $\beta_{Pop}^{k},\;k\;\epsilon \{1,\ldots ,K\}$ to predict selection probabilities *knowing the state*. Finally, state-specific Spearman rank correlation (*r*_*s*_) for observed and random steps can be used to assess model predictive capability. To do so, we first split up the steps of the 20% non-sampled individual’s trajectory according to their predicted movement mode. Then for each step, we ranked observed and random steps according to their selection probability. For example, if one observed step is coupled with 20 random steps in a stratum, the observed step can thus obtain a rank between 1 and 21. Its rank will be equal to one if its selection probability is the highest among the 21, whereas it will be equal to 21 if its selection probability is the lowest. At last, Spearman rank correlation (*r*_*s*_) is performed between the possible ranks [e.g., 1–21] and its associated frequency, separately for observed and one randomly sampled random steps. Spearman rank correlation ranges between -1 and 1 such that if it equals to 1, it means that the ranks of the steps are all equal to 1 (i.e., step selection probabilities are always the highest), whereas if it equals to -1, it means that the ranks of the steps are all equal to 21 (i.e., step selection probabilities are always the lowest). Finally, if the Spearman rank correlation is equal to 0, it means that the ranks of the steps range uniformly between 1 and 21 (i.e., step selection probabilities vary uniformly between 0 and 1). A good predictive capability of the HMM-SSF would thus lead to Spearman rank correlation for observed steps close to 1, meaning that predicted step selection probabilities of observed steps are generally higher than predicted step selection probabilities of random steps. The Spearman rank correlation score for the one randomly sampled random steps should, on the other hand, be 0 or negative as it would reflect that predicted selection probabilities of random steps are generally lower than predicted selection probabilities of observed steps. The calculation of Spearman rank correlation can be repeated 100 times using each time a new 80%-20% sample of individuals, to calculate means and ranges of *r*_*s*_ for each movement mode and random or observed group.

### Simulation studies

#### Simulations based on state-specific step selection functions

We simulated animal movement in three ecological scenarios to illustrate the ability of the HMM-SSF to identify and correctly predict behavioral modes and habitat selection process in different ecological settings. Simulated individuals moved in a virtual landscape and behaved either as: 1. foragers moving among resource patches, 2. foragers moving among resource patches while being attracted to a distant target (thereafter called migrants), and 3. foragers moving among resource patches and escaping a predator. Agents moved 300 (scenario 2) or 500 (scenarios 1 and 3) steps in a heterogeneous landscape and according to a state-specific step selection function. Two movement modes were defined: an encamped mode (*k* = 1) corresponding to foraging movement (or intra-patch) and a travelling mode (or inter-patch, *k* = 2). In scenario 3, one predator was also moving in the landscape according to a single-state SSF.

We randomly dispatched 500 resource patches of 25 ha over a 700 km^2^ surface (see the R code provided on Open Science framework https://osf.io/v5pnc/). We set patch quality by drawing an integer value between 1 and 10 independently for each patch. Quality outside resource patch was set to 0. A target was placed at the center top of the map for the migration scenario. The target could, for example, reflect the centroid of a previously visited calving area that individuals tend to aim at while migrating (i.e., site fidelity).

Movement mode of the forager was set at each step according to individual’s location: encamped if it was in a resource patch, travelling otherwise. In scenario 3 (i.e., presence of a predator), the same rule applied except that if the predator was near the forager (i.e., ≤ 500 m), the movement mode of the forager was set to travelling, independently of the cover type. By assigning transition rules based on environmental covariates or predator proximity, we violated the HMM-SSF assumption that the transitions in movement modes are governed by a Markov chain. Indeed, the HMM-SSF estimates movement modes and habitat selection behavior associated with these modes assuming that the transitions are governed by a Markov chain, i.e., by constant transition probabilities [27]. However, it is not representational of transition rules observed in nature since transition probabilities can depend on covariates varying in space and through time [30]. We thus simulated individuals moving according to a two-state step selection functions for which transition rules were, more realistically, based on environmental covariates. We did so to assess how robust the HMM-SSF can be to correctly identify movement modes and correctly infer the underlying selection processes while not accounting for the spatio-temporal variability in transition probabilities.

For each simulation, an individual’s starting point was randomly drawn either on the whole map (scenarios 1 and 3), or in the southern portion of the map (scenario 2). Then, 20 random locations were drawn within a disk of 2 km radius, centered on the individual’s current position. Landscape attribute (i.e., patch quality at random location) and step characteristics (i.e, step length and step direction) were extracted for each of the 20 available steps. The direction toward target from the individual’s current location was also calculated for the migration scenario. Then, a state-specific probability of being selected was assigned to each available step *i ϵ* {1,…20} using,

$$P_{i}^{k}=\frac{\exp\left(A_{i}^{k}\right)}{\sum_{i=1}^{20}\exp\left(A_{i}^{k}\right)},$$ (6)

where

$$A_{i}^{k}=\beta_{DP}^{k}∙\cos\left(\Delta_{DP}\right)+\beta_{SL}^{k}∙{SL}_{i}+\beta_{log.(SL)}^{k}∙\log\left({SL}_{i}\right)+\beta_{Q}^{k}∙Q_{i}+\beta_{T}^{k}∙\cos\left(\Delta_{T}\right),$$ (7)

where *k* = 1 if the state of the agent was encamped or *k* = 2, if the state of the agent was travelling, Δ_*DP*_ is the directional persistence (turning angle), *SL*_*i*_ is the length of step *i*, *Q*_*i*_ is the patch quality at the end of step *i* and Δ_*T*_ is the difference between the angles of the direction of the target and the direction of step *i*. Vectors of values for the scenario- and state-specific selection coefficients, ***β***^***k*,*scenario***^, *k ϵ* {1, 2}, *scenario ϵ* {*forager*, *migrant*, *forager with predator*} were set as follows (same order as in Eq 7): ***β***^**1,*forager***^ = [0.0; −3.0; −0.5; 1.0; *NA*]; ***β***^**2,*forager***^ = [1.0; −0.3; −0.05; 0.0; *NA*]; ***β***^**1,*migrant***^ = [0.0; −3.0; −0.5; 1.0; 0.0]; ***β***^**2,*migrant***^ = [1.0; −0.3; −0.05; 0.0; 1.0]; ***β***^**1,*forager with predator***^= [0.0; −3.0; −0.5; 1.0; *NA*]; ***β***^**2,*forager with predator***^= [1.0; −0.3; −0.05; 0.0; *NA*].

In scenario 3, two walkers were simulated: a forager following the same movement rules as presented above, and a predator tracking its prey resource. The predator had the same movement rules as its prey, except that only one movement mode was modelled using the following selection coefficients: *β*_*DP*_ = 1; *β*_*SL*_ = −0.3; *β*_log (*SL*)_ = −0.05; *β*_*Q*_ = 0.1. All simulations were implemented using R software (R code of the simulations is provided on Open Science framework https://osf.io/v5pnc/).

For each scenario, 500 repetitions were produced to estimate population level state-specific selection coefficients using 1) the HMM-SFF and 2) a 2-step approach. Specifically, the 2-step approach consisted of identifying the movement mode for each step using a multi-state correlated random walk and applying a single-state step selection function for each movement mode. We then contrasted 1) population level state-specific selection coefficients from the two approaches to the true parameters, 2) predicted states from the two approaches to the true states and 3) model predictive performance using *k*-fold cross validation.

For each repetition, we first drew 20 random locations for each step along the individual’s trajectory, within a buffer of 2 km around observed locations. Then, we extracted the same covariates as in Eq 7 for both observed and random steps and used them to fit an HMM-SSF to each individual’s trajectory. For each scenario, we calculated population level state-specific selection coefficients and their standard errors using Eqs 4 and 5. We used a receiver-operating characteristic (ROC) curve to compare true and predicted states. For the travelling state for example, each step obtained both the value of 1 if the true state was travelling or 0 otherwise, and the predicted conditional probability to be in travelling state from the HMM-SSF, which were then contrasted in the ROC curve. Finally, we used the same scheme as presented in section **Evaluation of model predictive capability** to perform the *k*-fold cross validation.

For each scenario, we fitted a 2-state correlated random walk (HMM-CRW) to the 500 individual trajectories. A 2-state correlated random walk assumes that individual trajectories are a combination of two correlated random walks, each having state-specific parameters for turning angle and step length distributions [6]. Each correlated random walk implies correlation in movement directionality, which can be positive (e.g., when moving straight) or negative (e.g., moving back and forth). Movement modes were modelled using a 2-state hidden Markov chain and transition probabilities between movement modes depended on environmental covariates [31]. We chose the commonly used gamma and von Mises distributions for step length and turning angle, respectively. We let the transition probabilities depend on patch quality for the forager and migrant scenarios, and on both patch quality and the natural logarithm of wolf distance for the forager with predator scenario. We implemented the HMM-CRW using the *moveHMM* package in R software [30]. We used, a posteriori, the *stateProbs* function of *moveHMM* to decode the probability of being in each movement mode at each step of the individual trajectories [30]. We used a ROC curve to compare true and predicted states from the HMM-CRW.

For the second part of the analysis, we first dichotomized the probabilities of being in travelling and encamped mode to 0–1 using a 0.50 threshold and assigned each step to the encamped or travelling group according to their dichotomized value. Then, we performed a single-state SSF to each movement mode. We drew 20 random locations for each step of each group along individual’s trajectory, within a buffer of 99^th^ percentile of state-specific step length distribution around observed locations (between 0.670 km and 0.750 km for the encamped state, 2.0 km for the travelling state). Then, we extracted the same covariates as in Eq 7 for both observed and random steps and used them to fit the single-state SSFs. We used robust estimates of the variance of selection coefficients to calculate their 95% confidence intervals [500 independent clusters, each being composed of the data of one individual, 32]. Finally, we performed *k*-fold cross validation developed for single-state SSF [31], for each movement mode.

#### Simulations based on multi-state biased correlated random walk

We evaluated whether the HMM-SSF successfully identifies movement modes and state-specific habitat selection when simulation assumptions are different from the underlying assumptions of the HMM-SFF. To do so, we used the simulation framework developed by [33] to simulate correlated biased random walkers in heterogeneous landscapes. Their model simulates an animal grazing across stationary resources that deplete and regenerate, and is based on three processes: consumption and regeneration of resources, a walker’s resource memory, and state-specific biased correlated movement process.

The instantaneous rate of change in habitat quality is modelled as:

$$\frac{dQ}{dt}(z,t)=(R(z,t)-C(z,t))Q(z,t),$$ (8)

where *Q*(*z*, *t*) represents the habitat quality at location *z* and time *t*, *R* and *C* are the regeneration and consumption functions, respectively. More precisely,

$$R(z,t)=\beta_{R}\left(1-\frac{Q(z,t)}{Q(z,0)}\right)$$ (9)

with *β*_*R*_ being the regeneration rate, and

$$C(z,t)=\beta_{C}f_{c}(|z-Z|),$$ (10)

with *β*_*C*_ being the consumption rate, *f*_*c*_ an isotropic spatial kernel with bivariate normal distribution ($N_{2}(0,\gamma_{C}^{2}I)$) and *Z* the animal’s position. Consumption is maximal at the animal’s position, i.e., when *z* = *Z*, but also occurs more or less widely in the vicinity of the animal depending on the value of $\gamma_{C}^{2}$.

The memory map (*M*(*z*, *t*)) of the animal works as a combination of a long-term (*L*(*z*, *t*), attractive effect) and a short-term (*S*(*z*, *t*), repulsive effect) memory. Specifically, the instantaneous rate of change in the long-term memory is modelled as:

$$\frac{dL}{dt}=\beta_{L}f_{L}(\left|z-Z\right|)(Q_{0}-L)-\phi_{L}L,$$ (11)

where *β*_*L*_ is the learning rate of the long-term memory, *f*_*L*_ an isotropic spatial kernel with bivariate normal distribution ($N_{2}(0,\gamma_{L}^{2}I)$) and *ϕ*_*L*_ is the decaying rate of the long-term memory. The instantaneous rate of change in the short-term memory is similarly modelled as:

$$\frac{dS}{dt}=\beta_{s}f_{S}(\left|z-Z\right|)(Q_{0}-S)-\phi_{S}S,$$ (12)

where *β*_*S*_ is the learning rate of the short-term memory, *f*_*S*_ an isotropic spatial kernel with bivariate normal distribution ($N_{2}(0,\gamma_{S}^{2}I)$) and *ϕ*_*S*_ is the decaying rate of the short-term memory. Finally, the attractive effect of the long-term memory and the repulsive effect of the short-term memory are integrated in the memory map using,

$$M=L-\psi_{M}S.$$ (13)

*M* can thus vary between negative (i.e., repulsive effect) and positive (i.e., attractive effect) values. *ψ*_*M*_ is used to makes *S* decaying faster than *L* such that a just-visited location will have a *M* negative value.

The movement process of the animal is determined according to one of the two behavioral states it can be in: either feeding (i.e., encamped) or searching (i.e., travelling). The individual switches from feeding mode to searching mode when its instantaneous rate of consumption drops below the average consumption rate, and inversely. In the encamped mode, the individual moves according to a continuous correlated random walk. In the travelling mode, the individual moves according to a continuous biased correlated random walk, for which the bias is determined from the memory map weighted by a spatial kernel of distance with exponential distribution (Exp(*γ*_*z*_)), such that the animal searches for known productive patches that are also close [see 33]. The autocorrelation in movement direction (*τ*) is stronger in the searching mode than in the feeding mode (*τ*_*F*_>*τ*_*S*_), and the individual also moves faster when searching than feeding (*v*_*F*_>*v*_*S*_).

We generated three landscapes of 50×50 cells with different levels of patchiness using a gaussian random field (S1 Fig). We used an exponential covariance function with variance = 1, nugget = 0 and a set of patch concentration (*μ*_*Q*_) and patch size (*γ*_*Q*_) to obtain three level of patchiness: low (*μ*_*Q*_ = -1.5, *γ*_*Q*_ = 2), intermediate (*μ*_*Q*_ = -0.5, *γ*_*Q*_ = 2) and high (*μ*_*Q*_ = 1, *γ*_*Q*_ = 10) (S1 Fig). Following [33], negative values were truncated to 0 and landscapes were normalized to sum to one. The *RandomFields* R package was used to produce the landscapes [34]. Each landscape was associated to one scenario and 500 repetitions were produced for each scenario. Model parameters for each scenario were initialized based on [33] and are reported in S1 Table. The continuous time model was implemented in Java, with time discretized with small regular intervals Δ*t* approximating *dt*. Simulation duration was fixed to 500 time steps.

For each repetition, we first drew 20 random locations for each step along the individual’s trajectory, within a buffer of 99^th^ percentile of scenario-specific step length distribution around observed locations. Then, for both observed and random steps, we extracted the following covariates: the cosine of step turning angle, the step length, the natural logarithm of step length, the patch quality at the end of the step and for the high and intermediate patchiness scenarios the distance to closest habitat patch. We then used those covariates to fit an HMM-SSF to each individual’s trajectory. We used the distance to habitat patch because individuals could feed even though they were outside of resource patch due to the continuous consumption process implemented with the spatial kernel *f*_*c*_ (Eq 10). For each scenario, we calculated population level state-specific selection coefficients and their standard error using Eqs 4 and 5. We used a ROC curve to compare true and predicted states. Finally, we used the same scheme as presented in section **Evaluation of model predictive capability** to perform the *k*-fold cross validation. As true parameter values for the state-specific step selection functions were unknown, we could not compare the estimates to the true values.

### Case studies

#### Ethical statement

The investigation was carried out in compliance with the institutional ethical standards and norms in force. Permits for bison and wolf research came from the Ethical Committee Comité de protection des animaux de l’Université Laval (#2017001–1) and the research permit came from Parks Canada Agency (PA- 2016–21697). Mule deer permit was provided by the Wyoming Game and Fish Department. The zebra research was authorized by the Zimbabwe Parks and Wildlife Management (permit numbers: REF:DM/Gen/(T) 23(1)(c)(ii): 03/2009, 01/2010, 25/2010, 05/2011, 06/2011, 12/2012, 15/2012, 08/2013).

#### Identifying the onset of migration and habitat selection during the different phases of the migratory behavior

**Fig 1:**
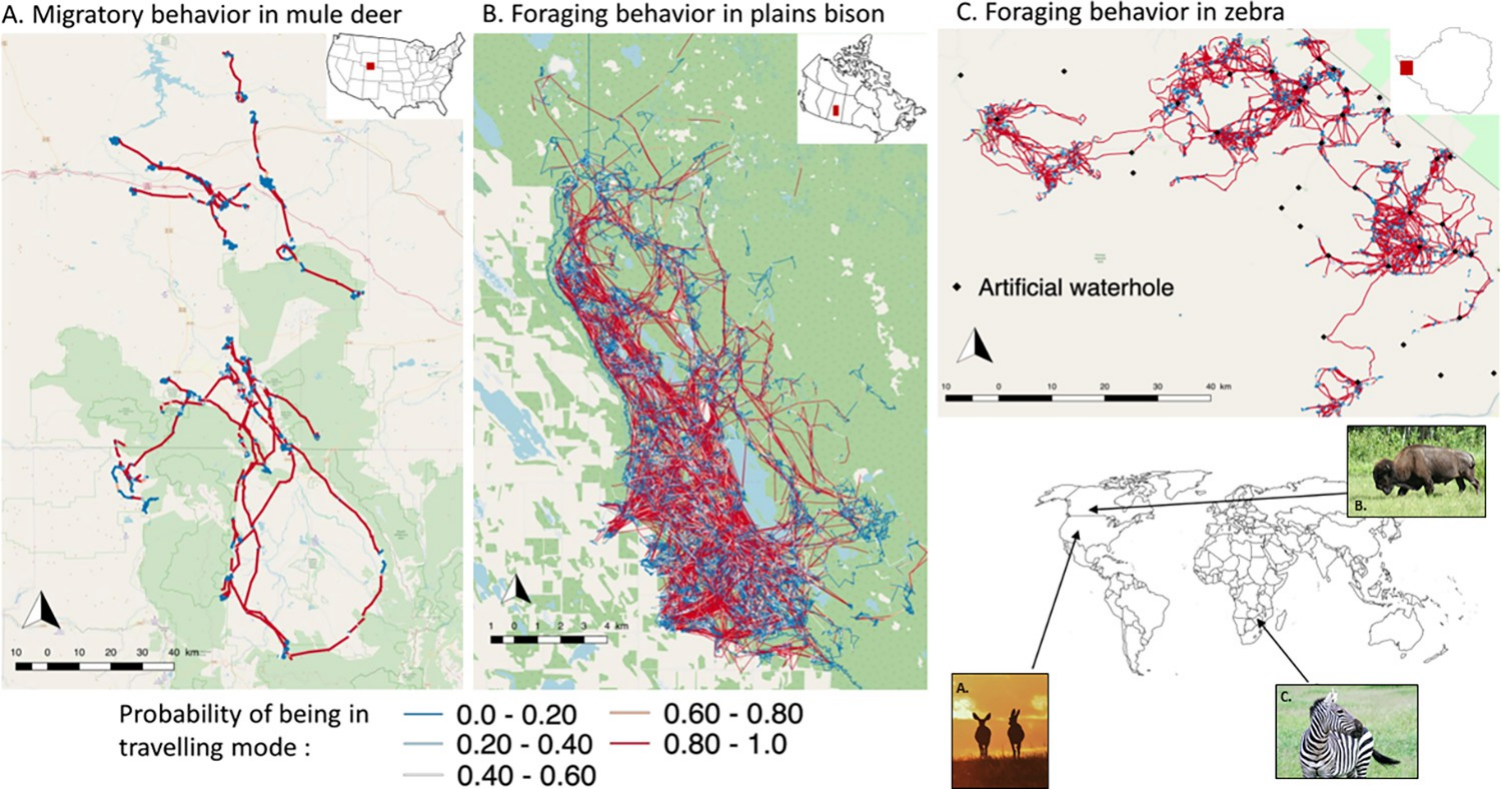
Trajectories of 42 plains bison geolocalised every hour between 2005 and 2016, during summer season in Prince Albert National Park (SK, Canada), 15 mule deer geolocalised every 3 hours during 2012 spring migration in and around Medicine Bow National Forest (WY-CO, USA) and 17 zebras geolocalised every half hour between 2012 and 2014, during dry hot season in Hwange National Park (Zimbabwe). The conditional probability of being in travelling mode of movement at each step, estimated from the HMM-SSF, is represented using a blue (low) to red (high) gradient.

We used GPS locations of 15 mule deer relocated every three hours from 2011 to 2012 in Medicine Bow National Forest (MBNF, Wyoming-Colorado, USA, Fig 1). In total, 13,444 relocations were used in the analysis. The study area is located in a semi-arid mountainous region, mainly covered with shrub (47%), coniferous or deciduous forest (32%) and herbaceous grassland or wetlands (18%). In winter, deer range in valleys around MBNF then migrate during spring (between April and June) to spend summer at higher elevation of MBNF (Fig 1), then return back to lower elevation during fall (between October and December).

We used landscape attributes known to influence mule deer movements during spring migration. Plant productivity and biomass in MBNF was depicted with the Integrated Normalized Difference Vegetation Index (INDVI), calculated using NDVI time series from 2012 MOD09Q1 data product [35]. Canopy cover (%) was obtained from the 2011 National Land Cover Database (30 m resolution), aspect (ranging from -1 as southerly to 1 as northerly aspects), and slope degree were derived from the US Geological Survey National Elevation Dataset [35]. To evaluate whether deer oriented their 2012 migration movement toward summer area visited in 2011, we identified the centroids of their GPS locations while they were on their summer range in 2011. To do so, we plotted the net squared displacement (NSD) of each individual over time, and manually identified the end of spring migration and the start of fall migration [see Appendix S3 of 36]. We used geolocations within this time interval to calculate 2011 summer range centroid for each individual. On average, 585 geolocations (range: 181–1165) were observed within 2011 summer range.

Mule deer data included 2012 geolocations that were collected from one week prior spring migration to one week after the end of spring migration. We then built individual trajectories using successive GPS-locations at three hours intervals. We drew 20 random locations for each observed step, within a buffer around the location at the start of the step. The buffer’s radius (i.e., 3.8 km) was determined using the 99^th^ percentile of the step length distribution obtained from the trajectories of all individuals combined. The radius was thus the same for all behavioral phases since we did not know a priori the distance travelled in each movement mode. By doing this, we might overestimate landscape attributes available to the individuals in the encamped mode, since they travel smaller distances in this movement mode. For each observed and random step, we then extracted, at locations at the end of the step: the aspect, the slope degree, the INDVI and the % of canopy cover. We also calculated the directional bias toward the 2011 deer summer range and computed step length and turning angle for both observed and random steps.

We used the conditional probability of being in travelling mode at each step, estimated from the HMM-SSF, to identify the start date of migration for each individual. Specifically, we first dichotomized the probability to 0–1 using a 0.50 threshold. Then, we identified the date of the first step in travelling mode. We then compared those dates to the date obtained from the NSD. By doing this, we did not evaluate whether the HMM-SSF can identify migration onset from an entire year but instead, whether it can assist in the determination of a more accurate start date from a coarse estimation.

#### Evaluating behavior-habitat relationship during movement modes of foragers

We used GPS relocations of 42 plains bison from 2005 to 2016 in Prince Albert National Park (Saskatchewan, Canada), and data from 18 zebras collected from 2012 to 2014 in Hwange National Park (Zimbabwe) (Fig 1). Bison were relocated every hour, whereas zebras were relocated every half hour. We based our analysis on 94,686 and 72,730 relocations for bison and zebras, respectively.

Bison occupy a relatively flat area composed of deciduous and conifer stands (85%), meadows (10%) and water bodies (5%). Zebras in Hwange National Park occupy an area dominated by bushlands (>60%), with small patches of grasslands and larger patches of woodlands. During the dry hot season (August through October), zebra strongly rely on waterholes artificially supplied with pumped groundwater.

For both bison and zebra, we included landscape attributes important for their foraging behavior. Specifically, we used a supervised classification of a SPOT-5 multispectral image (August 2008, 10 m resolution) to delimit meadows, forest, water bodies and roads in Prince Albert National Park [37]. Bushlands, grasslands and woodlands were delimited in Hwange National Park using an unsupervised classification of Landsat-7 ETM+ satellite images (August 2002, November 2002 and April 2003, 30 m resolution, [38]. Finally, we also considered the location of 67 artificial waterholes that are distributed within zebra range [39].

Bison and zebra data encompassed geolocations that were collected from May to August (summer season) and from August to October (dry hot season), respectively. We then proceeded similarly as for the mule deer system: we drew 20 random locations for each observed step, within a buffer around the location at the start of the step. The buffer’s radius (i.e., 1.6 km for bison and 1.3 km for zebra) was determined using the 99^th^ percentile of the step length distribution obtained from the trajectories of all individuals combined. For each observed and random step, we then extracted the system-specific covariates, at locations at the end of the step: for bison, four binary variables indicating whether the location fell within a meadow, a forest patch, a water body or a road; and for zebras, a categorical variable indicating whether the location fell within a bushland, a grassland or a woodland, and the Euclidean distance to the closest artificial waterhole. Finally, we also computed step length and turning angle, for both observed and random steps.

We first ran one HMM-SSF on each individual of every dataset. We considered that an individual could have two modes of movement, representing encamped (*k* = 1) and travelling (*k* = 2) modes. We included the different system-specific landscape covariates presented above to fit the individual HMM-SSF. Euclidean distance to waterhole was log-transformed and the bias towards previously visited summer range was included using the cosine of the difference between the direction toward the centroid of 2011 summer range and step direction. In addition, we include the cosine of step turning angle, together with step length (in km) and log(step length) to characterize movements in both modes [27]. Specifically, we used $\frac{1+\beta_{log.step\;length}^{k}}{{-\beta }_{step\;length}^{k}},\;k\;\epsilon \;\{1,2\},$ to calculate average travelled distance in each movement mode when $\beta_{step\;length}^{k}<0$ and $\beta_{log.step\;length}^{k}>-1$ as this yields a gamma distribution for distance (see S1 Appendix). However, when $\beta_{step\;length}^{k}>0$ or $\beta_{log.step\;length}^{k}<-1$, this is not the density of a gamma distribution anymore such that we used Metropolis algorithm to simulate 20,000 distances from the density corresponding to $\beta_{step\;length}^{k}$ and $\beta_{log.step\;length}^{k}$, and averaged the last 10,000 to obtain average travelled distance (see S1 Appendix).

For each population, we calculated state-specific coefficient estimates using the individual HMM-SSF estimates and Eq 4. Finally, we assessed each model’s predictive capability using *k*-fold cross validation as presented in section **Evaluation of model predictive capability**. Statistical analyses were conducted using R software [40]. The datasets, together with R code for fitting individual models, estimating population level parameters, conducting *k*-fold cross-validation, and using the Metropolis algorithm are provided on Open Science framework (https://osf.io/v5pnc/).

#### Assessing the circumstances over which a forager flees a predator

We assessed how the movements of a forager can be influenced by the presence of a predator by studying the environmental factors influencing the transitions from encamped to travelling mode of movement.

We used the conditional probabilities of being in encamped or travelling mode at each step given the observed data (i.e., $P(S_{t}=k|F_{t-1}^{0}),\;k\;\epsilon \;\{1,2\},\;t\;\epsilon \;\{1,\ldots ,T\}$; [27]. Let *Pthreshold* be such that when $P(S_{t}=1|F_{t-1}^{0})>Pthreshold$, the movement mode at step *t* is encamped. We assumed that there has been a transition from encamped to travelling mode of movement when $P(S_{t}=1|F_{t-1}^{0})>Pthreshold$ and $P(S_{t+1}=1|F_{t}^{0})\leq 1-Pthreshold$. We assumed that the animal remained encamped when $P(S_{t}=1|F_{t-1}^{0})>Pthreshold$ and $P(S_{t+1}=1|F_{t}^{0})>Pthreshold$. We performed the analysis using several *Pthreshold* values (i.e., 0.5; 0.6; 0.7; 0.8). Note, however, that more and more data are discarded from the analysis with increasing values of *Pthreshold*.

For both transition and non-transition, we used the location at the end of step *t*, which also corresponds to the location at the start of step *t*+1, to extract environmental factors that could influence transitions. We first performed the analysis using simulated data (i.e., scenario 3); that is for each location associated to a transition or a non-transition, we extracted patch quality and the Euclidean distance between the forager location and the predator location at the same time. Given the movement rules imposed to agents, the analysis should indicate that simulated foragers switch from encamped to travelling mode when patch quality is null or the predator is close (i.e., ≤ 500 m).

We then performed the analysis using the bison dataset: we assessed how wolves influenced bison movement in Prince Albert National Park, by using two indices of wolf space use computed from the GPS relocations of 17 adult wolves from 5 packs between 2007 and 2016. More specifically, we first used Brownian bridge movement kernels (UD) to calculate a relative long-term intensity of space use each year (for more detailed description of kernel computation [for more detailed description of kernel computation, see 41]. We restricted locations of bison to 95% of core territories of wolf packs and extracted kernel value for each retained location, serving as a proxy for the spatial pattern in relative risk of wolf encounter. Secondly, we used the Euclidean distance between a given collared bison and the nearest wolf every time a bison was relocated. To account for the potential effect of other environmental factors on bison movement, we also extracted the following information: we divided each day into three time periods (i.e., dawn and dusk: 03:00–06:59 and 16:00–21:59; day: 07:00–15:59; night: 22:00–02:59, see S4 Fig), and used it as an explanatory factor (3 levels) since bison movement can change through daytime [29]. Finally, we included a binary variable indicating whether the location fell within a resource patch for bison (i.e., a meadow).

We compared environmental factors related to transition (*Y* = 1) and non-transition (*Y* = 0) using a generalized linear mixed-effects model with binomial distribution. We included a random intercept for each individual’s ID to control for the non-independence and the unbalanced design in the number of observations per individual. Model covariates included period of day, meadow, wolf kernel, distance to nearest wolf (in km) and the interaction between wolf kernel and wolf distance. Because wolf presence should have an effect on bison movement solely when they are in close vicinity, we used a truncated index of wolf distance (*d*_*wolf*_) calculated as follows,

$${\tilde{d}}_{wolf}=\left\{ \begin{array}{c} d_{wolf},\;if\;d_{wolf}\leq d_{threshold}\\ d_{threshold},\;otherwise \end{array}\right.$$ (14)

and used ${\tilde{d}}_{wolf}$ as model covariate instead. We ran 50 models for which we varied *d*_*threshold*_ from 0.1 km to 5 km, and identified the optimal *d*_*threshold*_ by investigating the log-likelihood profile. Statistical analyses were conducted using the *lmerTest* package in R software [40].

We also estimated state-specific selection coefficients for the bison population using a 2-step approach similarly to the one presented in section **Simulation studies** > **Simulations based on state-specific step selection functions** > **Statistical analysis**. Specifically, we fit a 2-state correlated random walk to all bison trajectories with Gamma and von Mises distributions for step length and turning angle, respectively. We let the transition probabilities depend on the following covariates (presented in section **Potential environmental factors influencing transition from encamped to travelling mode**): the period of day, the binary variable meadow, the wolf kernel, the natural logarithm of distance to nearest wolf and the interaction between wolf kernel and the natural logarithm of wolf distance. The natural logarithm transformation should mimic the truncated index of wolf distance (Eq 14). Then, we performed a single-state SSF to each movement mode. We drew 20 random locations for each step of each group along each individual’s trajectory, within a buffer of 99^th^ percentile of state-specific step length distribution around observed locations (0.14 km and 2.0 km for the encamped and travelling state, respectively). Then, we extracted the same covariates as for the bison HMM-SSF and used them to fit the single-state SSFs. We used robust estimates of the variance of selection coefficients to identify the significant covariates (42 independent clusters each composed of the data of one individual, 32). Finally, we performed *k*-fold cross validation for both single-state SSFs [29].

## Results

### Comparison of the HMM-SSF and the 2-step approach performance using simulations

**Fig 2:**
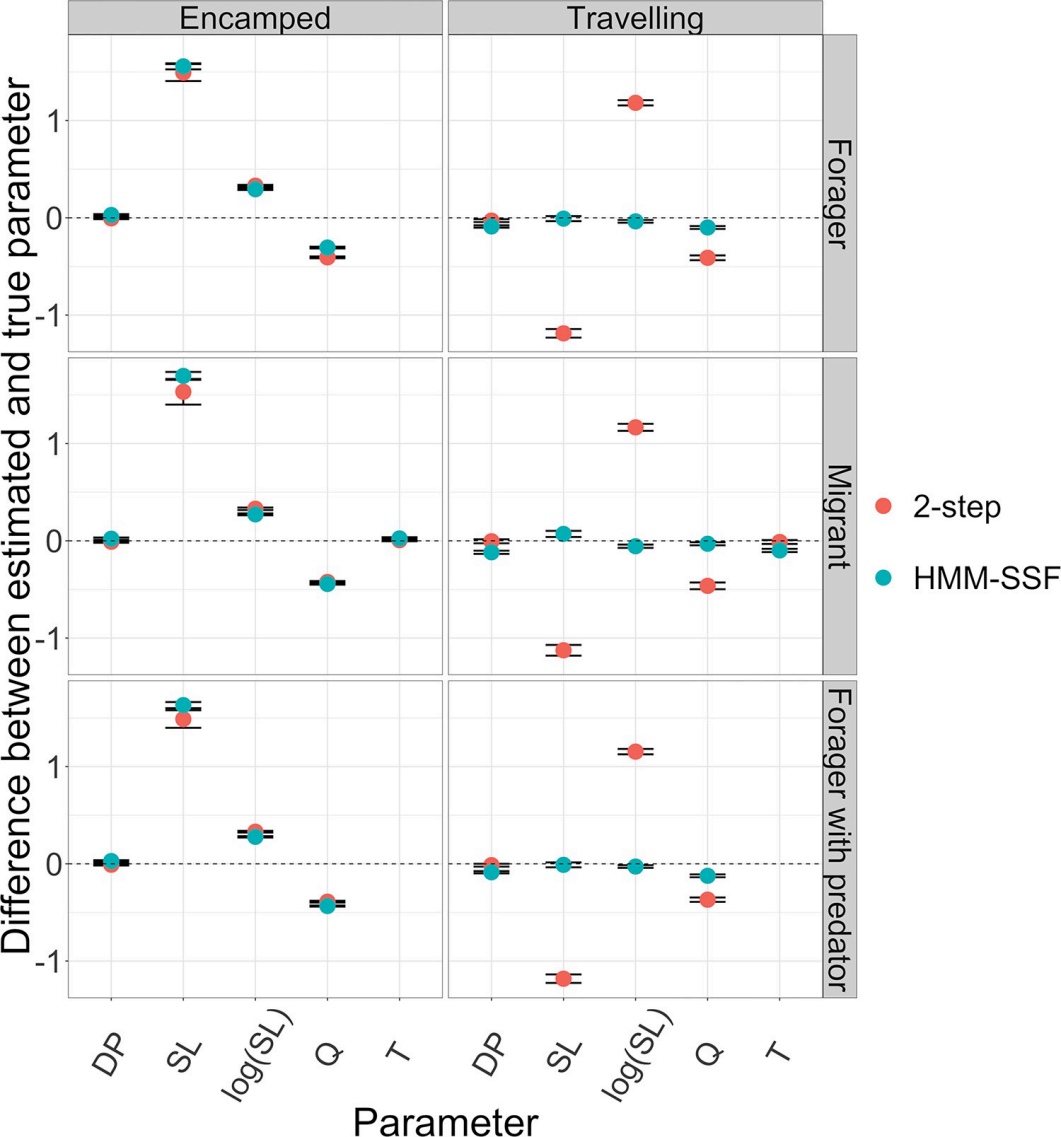
Difference between population level state-specific selection coefficients and their true parameters used to simulate individual’s trajectory, from state-specific step selection functions, in three ecological scenarios. Estimation from the HMM-SSF are represented in blue and estimation from the 2-step approach in red. 95% confidence interval of the difference is also shown with error bars. For each scenario, 500 trajectories were simulated from state-specific rules of movement and used to estimate population level state-specific selection coefficients. DP = directional persistence (cosine), SL = step length, log(SL) = natural logarithm of step length, Q = patch quality and T = target (see Eq 7).

Both approaches successfully identified true movement modes from the simulated individual trajectories as the area under the ROC curve were all higher than 0.97 for the HMM-SSF and the HMM-CRW, in all three scenarios (${AUC}_{HMM-SSF}^{Forager}=0.98;\;{AUC}_{HMM-SSF}^{Migrant}=0.97;\;{AUC}_{HMM-SSF}^{Forager\;with\;pred.}=0.98;\;{AUC}_{2-step}^{Forager}=0.98;\;{AUC}_{2-step}^{Migrant}=0.98;\;{AUC}_{2-step}^{Forager\;with\;pred.}=0.98$). The simulation analysis also showed that both the HMM-SSF and the 2-step approach performed relatively well at predicting habitat selection along the trajectory of simulated individuals (Table 1). Indeed, *k*-fold cross-validation indicated that models yielded accurate predictions of habitat selection for each movement mode, in the sense that selection probabilities estimated from either the HMM-SSF or the 2-step approach for the observed steps were higher than selection probabilities estimated for random steps, in all three scenarios (Table 1). However, estimation of population level state-specific selection coefficients for the travelling phase varied between the HMM-SSF and the 2-step approach, in all three scenarios (Fig 2). While the HMM-SSF provided rather similar selection coefficient estimates for the encamped phase than the 2-step approach, the HMM-SSF outperformed the 2-step approach for the estimation of some model parameters in the travelling state. Indeed, selection coefficient estimates of step length, its natural logarithm and patch quality for the travelling phase were closer to the true parameters from the HMM-SSF than from the 2-step approach (Fig 2).

**Table 1:** Assessment of the HMM-SSF predictive capability in comparison to a 2-step approach from simulations based on state-specific step selection functions, to predict habitat selection along encamped and travelling movement in three ecological scenarios, using *k*-fold cross validation. Means, minimums and maximums ([min; max]) of 100 Spearman rank correlation are reported for each movement mode and both observed and random steps.

Scenario			Encamped	Travelling
Forager	Observed	HMM-SSF	0.99[0.96; 1.0]	0.99[0.99; 1.0]
		2-step	0.98[0.97 ; 1.0]	0.99[0.99 ; 1.0]
	Random	HMM-SSF	-0.59[-0.91; -0.26]	-0.63 [-0.84; -0.18]
		2-step	0.01[-0.59 ; 0.57]	0.01[-0.52 ; 0.63]
Migrant	Observed	HMM-SSF	0.97[0.91; 1.0]	0.99[0.99; 1.0]
		2-step	0.98[0.96 ; 0.99]	0.99[0.99 ; 1.0]
	Random	HMM-SSF	-0.58[-0.80; -0.30]	-0.65[-0.93; -0.32]
		2-step	-0.05[-0.63 ; 0.54]	-0.03[-0.56 ; 0.55]
Forager with predator	Observed	HMM-SSF	0.99[0.97; 1.0]	0.99[0.99; 1.0]
		2-step	0.98[0.97 ; 0.99]	0.99[0.99 ; 1.0]
	Random	HMM-SSF	-0.62[-0.84; -0.17]	-0.65[-0.86; -0.23]
		2-step	-0.01[-0.57 ; 0.58]	0.04[-0.55 ; 0.66]

### HMM-SSF performance under multi-state biased correlated random walk simulations

Despite simulations from the multi-state biased correlated random walks differ from HMM-SSF assumptions, the HMM-SSF still successfully identified true movement modes from the simulated individual trajectories as the area under the ROC curve were all higher than 0.75, in all three scenarios of landscape patchiness (${AUC}_{HMM-SSF}^{Low\;patchiness}$ = 0.75; ${AUC}_{HMM-SSF}^{Interm.\;patchiness}=0.79;\;{AUC}_{HMM-SSF}^{High\;patchiness}=0.78$). *k*-fold cross-validation also indicated that models yielded accurate predictions of habitat selection for each movement mode, in the sense that selection probabilities estimated from either the HMM-SSF were higher than selection probabilities estimated for random steps, in all three scenarios (Table 2). Finally, the HMM-SSF identified different selection behaviors that were in accordance with the state-specific movement rules used to simulate individual trajectories, in all three scenarios. Indeed, individuals selected patches of high quality in the travelling state (i.e., when searching), whereas they were closer to resource patches in the encamped state (i.e., when feeding), for both the intermediate and high levels of patchiness (Table 2). For the low level of patchiness, the landscape was composed of one large resource patch covering almost the whole map (S1 Fig), such that simulated individuals remained most of the time in this resource patch. Still, individuals selected locations of high resource quality within that patch, but the selection was even stronger in the travelling state than in the encamped state, according to the HMM-SSF (Table 2).

**Table 2:** State-specific selection coefficient estimates along with their standard-error (SE) and associated P-value (P) of the HMM-SSF used to predict simulated animal movements according to encamped or travelling mode, in landscapes with varying levels of patchiness. For each scenario, 500 trajectories were simulated from multi-state correlated random walk and used to estimate population level state-specific selection coefficients. Means ($\bar{r_{s}}$), minimums and maximums ([min; max]) of 100 Spearman rank correlation are reported for each movement mode and both observed and random steps. Average travelled distances were calculated using step length and log-transformed step length coefficient estimates (see S1 Appendix).

Scenario		Encamped			Travelling		
Low patchiness	**Selection coefficient**	Estimate	SE	P	Estimate	SE	P
	*cos(Dir*.*pers)*	0.91	0.01	< .01	3.52	0.02	< .01
	*Step length*	-5.59	0.03	< .01	-1.22	0.01	< .01
	*log(Step length)*	3.49	0.02	< .01	1.99	0.02	< .01
	*Patch quality (x100)*	5.24	0.32	< .01	23.19	0.29	< .01
	***k*-fold ($\bar{r_{s}}$) [min-max]**						
	*Observed*	0.98 [0.96; 0.99]			0.83 [0.75; 0.91]		
	*Random*	-0.65 [-0.90; -0.30]			-0.68 [-0.90; -0.38]		
	**Average travelled speed (in cells/step)**	0.80			2.45		
Intermediate patchiness	**Selection coefficient**	Estimate	SE	P	Estimate	SE	P
	*cos(Dir*.*pers)*	0.91	0.01	< .01	3.42	0.02	< .01
	*Step length*	-5.35	0.03	< .01	-0.89	0.01	< .01
	*log(Step length)*	2.42	0.02	< .01	1.53	0.02	< .01
	*Patch quality (x100)*	-0.16	0.03	< .01	1.29	0.04	< .01
	*Distance to closest resource patch*	-0.80	0.02	< .01	-0.47	0.01	< .01
	***k*-fold ($\bar{r_{s}}$) [min-max]**						
	*Observed*	0.98 [0.93; 1.0]			0.85 [0.80; 0.94]		
	*Random*	-0.62 [-0.90; -0.25]			-0.69 [-0.92; -0.39]		
	**Average travelled speed (in cells/step)**	0.64			2.84		
High patchiness	**Selection coefficient**	Estimate	SE	P	Estimate	SE	P
	*cos(Dir*.*pers)*	0.91	0.01	< .01	3.74	0.02	< .01
	*Step length*	-5.55	0.03	< .01	-0.93	0.01	< .01
	*log(Step length)*	2.50	0.02	< .01	1.69	0.02	< .01
	*Patch quality (x100)*	-0.17	0.01	< .01	0.44	0.02	< .01
	*Distance to closest resource patch*	-0.69	0.01	< .01	-0.48	0.01	< .01
	***k*-fold ($\bar{r_{s}}$) [min-max]**						
	*Observed*	0.99 [0.97; 1.0]			0.89 [0.83; 0.93]		
	*Random*	-0.63 [-0.93; -0.25]			-0.67 [-0.94; -0.39]		
	**Average travelled speed (in cells/step)**	0.63			2.89		

Overall, the HMM-SSF shows robustness to the misspecification of the underlying processes that generates movement modes and transitions such that it can be used to make valid inferences on the underlying selection processes associated to movement modes from different ecological behaviors. Besides, the HMM-SSF provides more reliable population level state-specific selection coefficients than a commonly applied 2-step approach (Fig 2).

### Case studies

The HMM-SSF estimated different selection coefficients for the two distinct movement modes, encamped and travelling, in all three ecological systems (Table 3). *K*-fold cross-validation indicated that models yielded accurate predictions of habitat selection for each movement mode, in the sense that selection probabilities estimated from the HMM-SSF for the observed steps were higher than selection probabilities estimated for random steps, in all three scenarios (Table 3).

**Table 3:** State-specific selection coefficient estimates along with their standard-error (SE) and associated P-value (P) of the HMM-SSF used to predict animal movements according to encamped or travelling mode, in three ecological systems. A. Habitat selection during the different phases of the migratory behavior, B. and C. Habitat selection during the different phases of the foraging behavior. Estimates of model parameters from a 2-step approach are also shown for the bison dataset (B). Analysis were performed using 13,444, 94,686 (50592 for the 2-step approach) and 72,730 geolocations from 15 mule deer, 42 plains bison and 18 zebras over 2 (2011–2012), 11 (2005–2016) and 3 (2012–2014) years of monitoring, respectively. The predictive capabilities of the HMM-SSF and the 2-step approach (B) were assessed using *k*-fold validation repeated 100 times, means and ranges of Spearman rank correlation ($\bar{r_{s}}$) are reported for each behavioral mode of movement and both observed and random steps. Average travelled distances were calculated using step length and log-transformed step length coefficient estimates (see S1 Appendix).

**A. Migratory behavior in mule deer**												
**State**	**Encamped**						**Travelling**					
**Selection coefficient**	Estimate		SE		P		Estimate		SE		P	
*cos(Dir*.*pers)*	0.18		0.04		< .01		0.61		0.10		< .01	
*Step length*	-2.85		0.11		< .01		0.50		0.08		< .01	
*log(Step length)*	-0.10		0.03		< .01		-1.03		0.06		< .01	
*Directional bias towards previously visited summer area (cos)*	0.19		0.05		< .01		1.19		0.11		< .01	
*Aspect*	-0.12		0.05		0.01		-0.02		0.09		0.83	
*INDVI*	-0.001		0.004		0.83		0.01		0.01		0.02	
*Slope degree*	0.02		0.004		< .01		0.04		0.01		< .01	
*Treecover*	-0.003		0.002		0.12		-0.01		0.004		0.08	
***k-*fold ($\bar{r_{s}}$) [min-max]**												
*Observed*	0.91 [0.83; 0.97]							0.86 [0.67; 0.95]					
*Random*	-0.31 [-0.72; 0.15]							-0.15 [-0.56; 0.43]					
**Average travelled speed (in km/h)**	0.11							0.35					
**Proportion of time spent in each state**	0.83							0.17					
**Mean and range of duration of each state (in hour)**	63.0 [3.0; 777]							14.5 [3.0; 102]					
**B. Foraging behavior in plains bison**													
**State**	**Encamped**							**Travelling**					
**Selection coefficients**	Estimate		SE		P		Estimate		SE		P	
	HMM-SSF	2-step	HMM-SSF	2-step	HMM-SSF	2-step	HMM-SSF	2-step	HMM-SSF	2-step	HMM-SSF	2-step
*cos(Dir*.*pers)*	0.05	-0.44	0.01	0.01	< .01	< .01	1.21	0.82	0.02	0.02	< .01	< .01
*Step length*	-3.68	-25.11	0.05	1.16	< .01	< .01	-0.40	-2.64	0.05	0.07	< .01	< .01
*log(Step length)*	-0.55	-0.20	0.01	0.03	< .01	< .01	-0.27	0.22	0.02	0.02	< .01	< .01
*Water body*	-2.23	-1.45	0.11	0.26	< .01	< .01	-2.03	-2.58	0.18	0.10	< .01	< .01
*Forest patch*	-0.54	-0.44	0.03	0.06	< .01	< .01	-0.23	-0.26	0.07	0.05	< .01	< .01
*Meadow*	0.31	0.09	0.03	0.06	< .01	0.09	1.27	0.71	0.07	0.06	< .01	< .01
*Road*	0.05	-0.07	0.07	0.20	0.46	0.72	1.17	0.76	0.10	0.08	< .01	< .01
	**HMM-SSF**			**2-step**			**HMM-SSF**			**2-step**		
***k*-fold ($\bar{r_{s}}$) [min-max]**												
*Observed*	0.99 [0.94; 1.0]			0.97 [0.96; 0.99]			0.98 [0.92; 1.0]			0.96 [0.93; 0.99]		
*Random*	-0.57 [-0.83; -0.15]			0.01 [-0.53; 0.51]			-0.42 [-0.80; -0.01]			-0.01[-0.68; 0.79]		
**Average travelled speed (in km/h)**	0.12			0.03			1.83			0.46		
**Proportion of time spent in each state**	0.83			-			0.17			-		
**Mean and range of duration of each state (in hour)**	10.0 [1.0; 95.0]			-			1.0 [3.0; 47.0]			-		
**Transition frequency**												
To encamped	0.05			-			-			-		
To travelling	-						0.05					
**C. Foraging behavior in zebra**												
**State**	**Encamped**						**Travelling**					
**Selection coefficient**	Estimate		SE		P		Estimate		SE		P	
*cos(Dir*.*pers)*	0.42		0.01		< .01		2.210		0.030		< .01	
*Step length*	-6.50		0.11		< .01		-2.135		0.072		< .01	
*log(Step length)*	-0.58		0.01		< .01		0.238		0.023		< .01	
*Landcover* *												
*Grassland*	0.27		0.03		< .01		0.825		0.033		< .01	
*Woodland*	-0.10		0.03		< .01		-0.228		0.040		< .01	
*Distance to closest waterhole (log)*	0.61		0.06		< .01		-1.020		0.029		< .01	
***k*-fold ($\bar{r_{s}}$) [min-max]**												
*Observed*	0.98 [0.96; 1.0]						0.98 [0.92; 1.0]					
*Random*	-0.51 [-0.80; -0.21]						-0.487 [-0.86; 0.05]					
**Average travelled speed (in km/h)**	0.13						1.16					
**Proportion of time spent in each state**	0.77						0.23					
**Mean and range of duration of each state (in hour)**	8.0 [0.5; 94.5]						2.0 [0.5; 18.5]					

*reference category is *bushland*

### Identifying the onset of migration and habitat selection during the different phases of the migratory behavior

**Fig 3:**
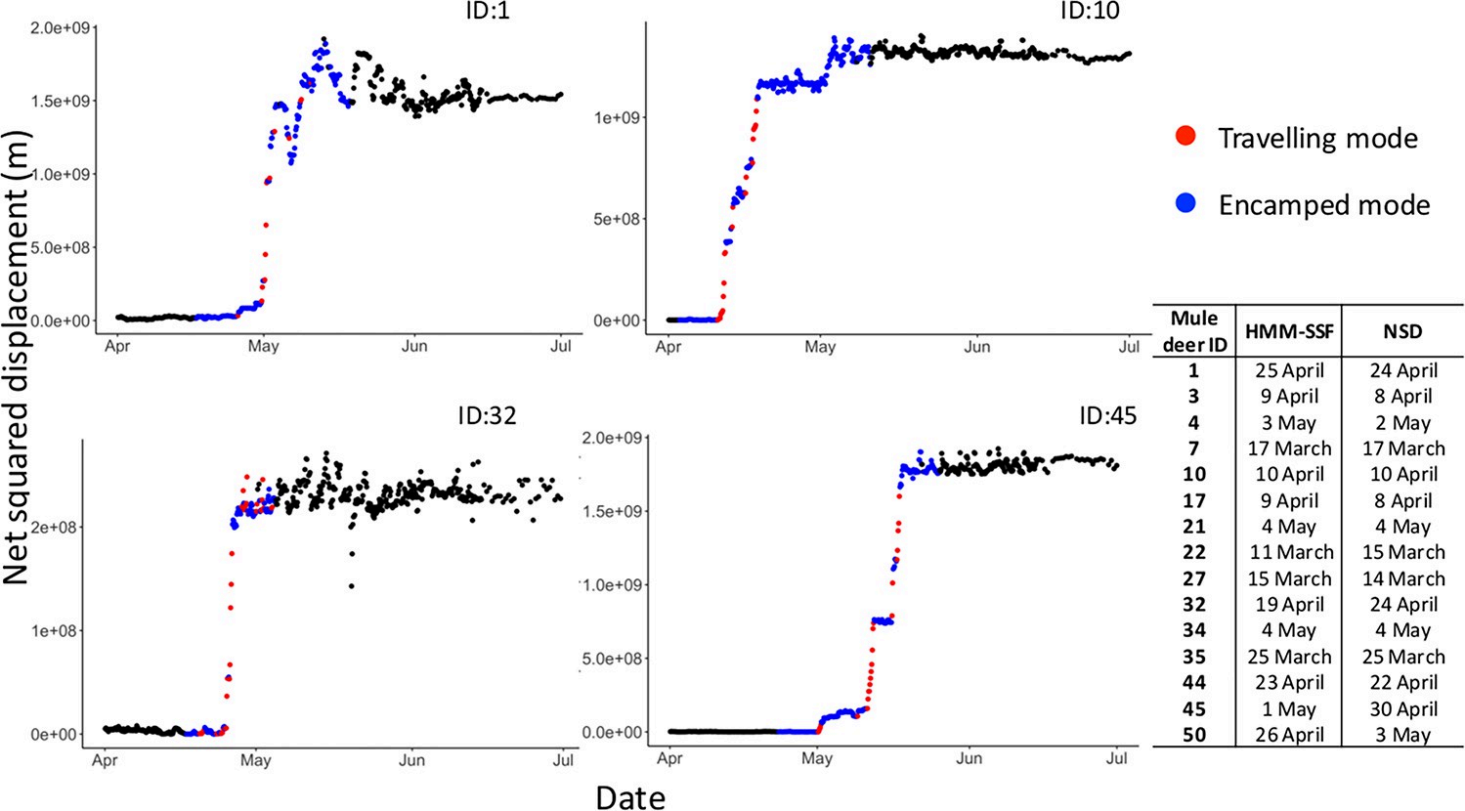
Net squared displacement over time for 4 mule deer geolocalised every 3 hours between April and July 2012 in Medicine National Bow Forest (WY-CO, USA). Net squared displacement is measured as the square of the Euclidean distance between location at a specific time t and start location (i.e., at time 1). Estimated mode of movement (red for travelling and blue for encamped) during the 2012 migration was based on the conditional probabilities of being in encamped and travelling mode at each step, as estimated from the HMM-SSF, and dichotomized to 0–1 using a 0.50 threshold.

The HMM-SSF identified the onset of mule deer migration between 11 March (first migration) and 4 May (last migration, Fig 3). Overall, the starting date of migration identified by the HMM-SSF was similar to that obtained by net squared displacement analysis (Fig 3), but the HMM-SSF provided information on selection behavior of individuals according to their movement modes.

**Fig 4:**
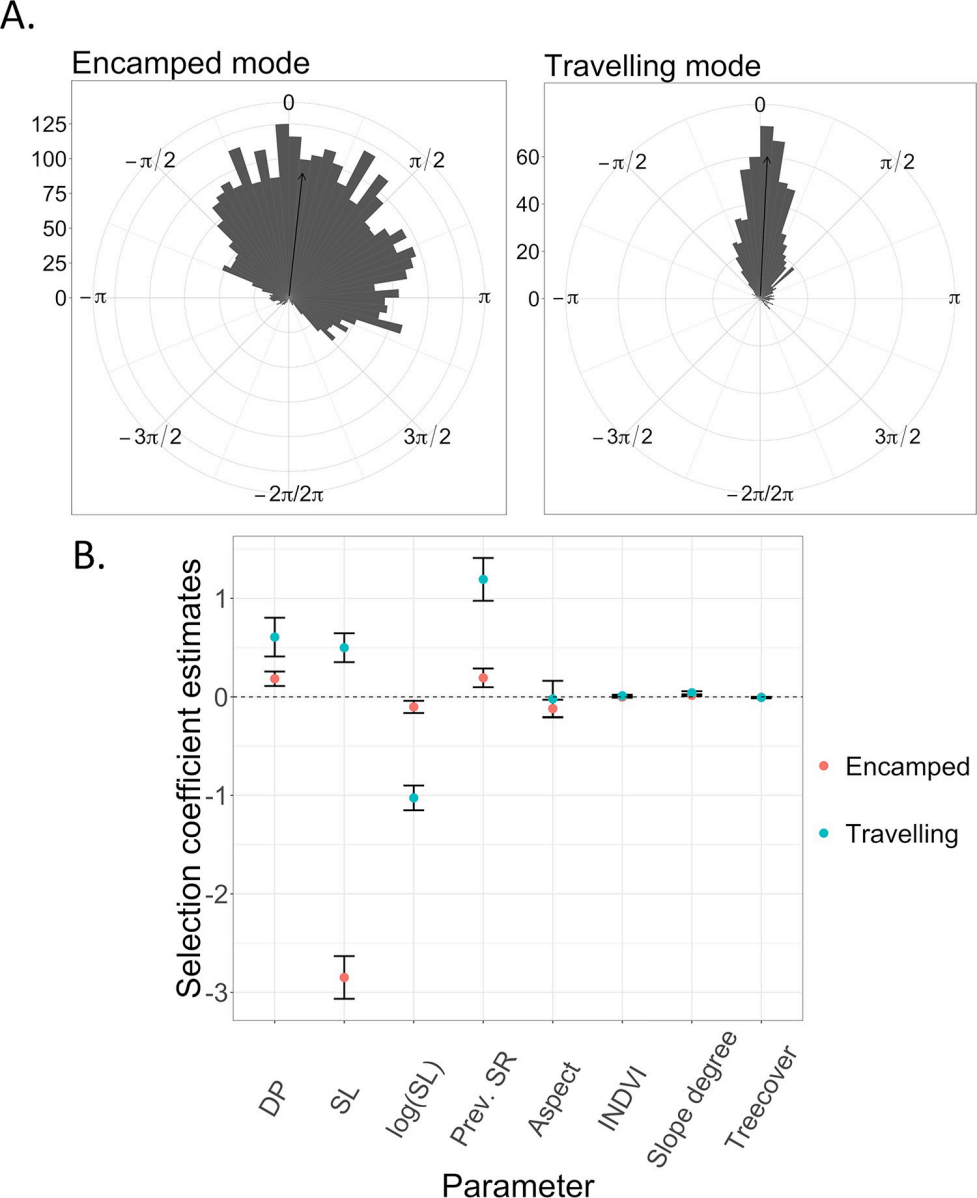
A. Distribution of directional bias (in radians) towards summer range visited in 2011 by mule deer in Medicine Bow National Forest, while they were migrating during spring 2012, according to the mode of movement estimated by the HMM-SSF at each step (either encamped or travelling). Estimated mode of movement was based on the conditional probabilities of being in encamped and travelling mode at each step, truncated to 0–1 using a 0.50 threshold. The arrows indicate the average directional bias. B. State-specific selection coefficient estimates from the HMM-SSF of different covariates influencing mule deer spring migration. DP = directional persistence (cosine), SL = step length, log(SL) = natural logarithm of step length, Prev. SR = directional bias towards previously visited summer ranger (cosine).

While migrating (i.e., travelling mode) mule deer moved toward the area they used the previous summer, at an average speed of 0.35 km/h (Table 3, Figs 3 and 4). During stopovers (i.e., encamped mode), they moved shorter distances (Table 3), but still with a slight bias towards their known summer range (Table 3, Fig 4). Deer selected biomass-rich locations while migrating whereas they selected southerly aspect locations during stopovers (Table 3, Fig 4). They also selected steep terrain, but the selection coefficient was larger for the travelling mode than for the encamped mode (Table 3, Fig 4).

### Evaluating behavior-habitat relationship during movement modes of foragers

As expected, both bison and zebras moved longer distances and in a more oriented way during inter-patch movements (i.e., travelling mode) than during intra-patch movements (i.e., encamped mode, Table 3, Fig 4). Moreover, selection coefficient estimates for resource patches (i.e., meadow for bison and grassland for zebra) were larger during inter-patch movements than during intra-patch movements, indicating that once they have left a patch they search for another (Table 3). Finally, bison selectively used roads to move among resource patches, whereas zebras selected locations close to nearby waterholes, along their way to another patch (Table 3, Fig 4 and S2 Fig).

#### Comparison to the 2-step approach applied to bison population

Although *k*-fold cross validation indicated that the 2-step approach yielded accurate predictions of habitat selection for each movement mode, some coefficient estimates from the 2-step approach were highly different, even opposed, to estimates from the HMM-SSF, in both movement modes (Table 3B). For example, in the encamped mode, the estimate of the directional persistence was positive from the HMM-SSF whereas negative from the 2-step approach, the estimates of step length was 8 times stronger from the 2-step approach than from the HMM-SSF and the estimates of meadow selection was positive for the HMM-SSF while not significant for the 2-step approach (Table 3B).

### Assessing the circumstances over which a forager flees a predator

The transition model revealed that the simulated foragers had a higher probability of switching from encamped to travelling mode when foragers were in a non-resource patch (i.e., patch quality = 0) and when simulated predators were in close vicinity of the foragers (S2 Table). Thus, the transition model correctly identified the behavior of fleeing from predators that was created in the simulation. In addition, the estimates from the transition model behaved similarly than the estimates from the HMM-CRW, which directly incorporates covariates in the transition probabilities of the Markov chain using a multinomial logit link (S2 Table).

**Fig 5:**
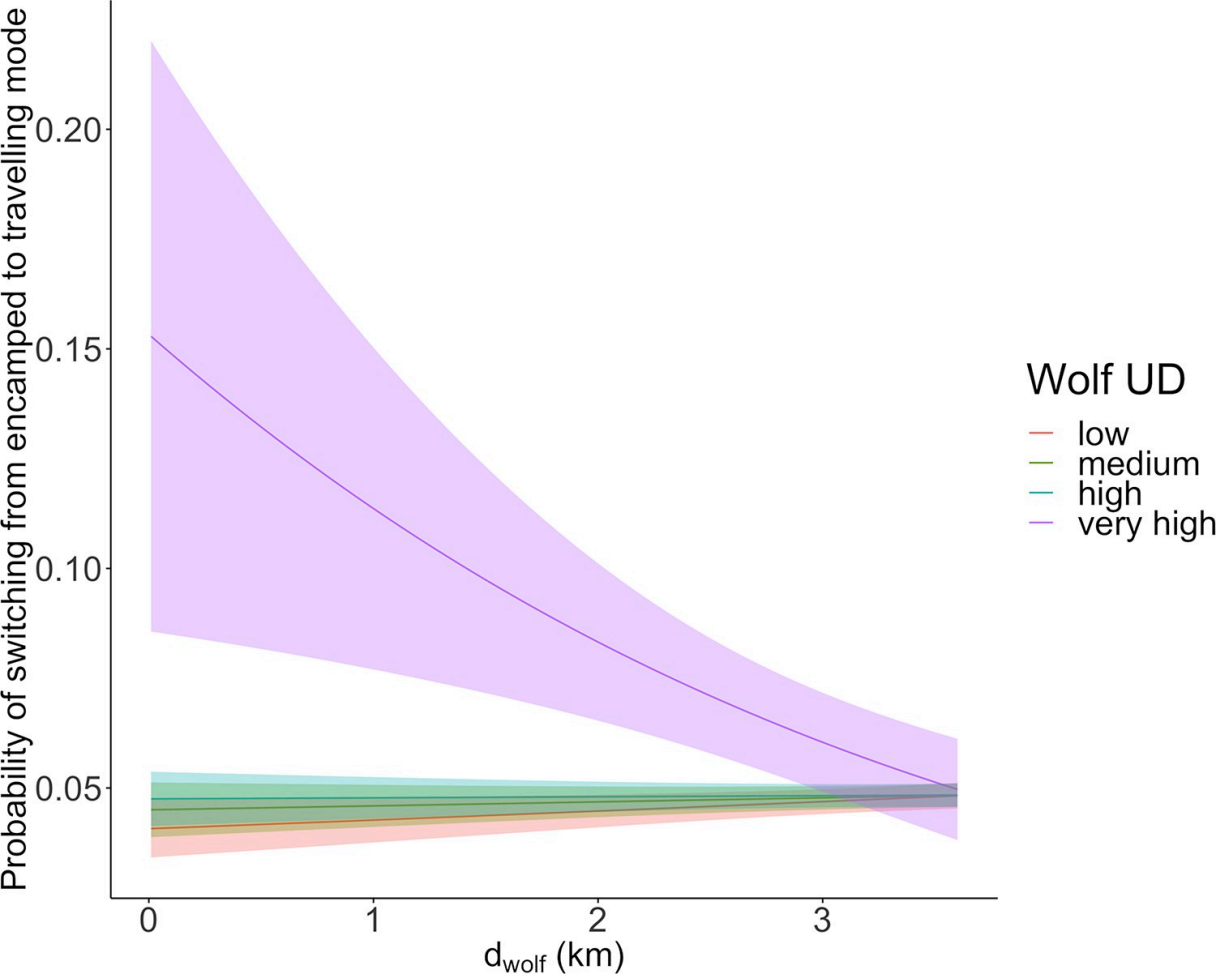
Estimated probability of switching from encamped to travelling mode of movement and 95% confidence interval, in plains bison during summer, according to wolf distance and local intensity of wolf utilization distribution (wolf UD). Coefficient estimates and standard errors were obtained from the fit of a mixed-effects generalized linear model with binomial distribution on 27,101 geolocations from 32 plains bison followed between 2007 and 2016 in Prince Albert National Park (SK, Canada). Probabilities were calculated here, by setting period of day and meadow variables to *day* and 1, respectively (see Table 3).

The movement response of bison to wolf presence was context dependent. Bison were more likely to change from encamped to travelling mode when in close vicinity of wolves together with a high relative risk of wolf encounter (i.e., high UD, Table 4 and S3 Table, Fig 5). This effect of wolf proximity in places of high wolf UD lessened gradually with distance from the nearest wolf, and vanished when bison were > 3.6 km from a wolf (Fig 5 and S3 Fig). Two other factors influenced the switch from encamped to travelling mode. First, a switch had higher probability to occur during dawn or dusk than during the day or at night (Table 4 and S3 Table). Second, bison had higher probability of switching to the travelling mode when they were outside resource patches (Table 4 and S3 Table). The estimates from the transition model also behaved also similarly than the estimates from the HMM-CRW (Table 4).

**Table 4:** Coefficient estimates along with their 95% confidence interval (95% CI) of the mixed-effects generalized linear model with binomial distribution (HMM-SSF + GLMM) and the multi-state correlated random walk model (HMM-CRW) to predict probability that a bison switched from encamped to travelling mode during its travel in summer. In the HMM-SSF + GLMM analysis, we used a probability threshold of 0.50 (*Pthreshold*) to determine transition and non-transition from the conditional probabilities of being in encamped or travelling state, as estimated from the HMM-SSF. Mixed-effects generalized linear model was fitted using 27,101 geolocations from 32 GPS-collared plains bison in Prince Albert National Park (SK, Canada). ${\tilde{d}}_{wolf}$ was set to the actual distance between bison and wolf (*d*_*wolf*_) when *d*_*wolf*_ ≤ *d*_*threshold*_ and *d*_*threshold*_, otherwise. *log*(*d*_*wolf*_) is the natural logarithm of *d*_*wolf*_.

Effect	Estimate		95% CI	
	HMM-SSF + GLMM	HMM-CRW	HMM-SSF + GLMM	HMM-CRW
** **				
*Intercept*	-2.92	-0.59	[-3.24; -2.60]	[-0.66; -0.51]
Period of day*				
*Dawn-Dusk*	0.53	0.17	[0.42; 0.64]	[0.11; 0.23]
*Night*	-1.51	-2.09	[-1.74; -1.28]	[-2.19; -1.98]
*Meadow*	-0.21	-0.11	[-0.32; -0.10]	[-0.16; -0.05]
*Wolf UD*	0.17	0.09	[0.05; 0.28]	[0.04; 0.14]
*${\tilde{d}}_{wolf}$*	0.04	**-**	[-0.04; 0.12]	-
*Interaction (Wolf UD,* ${\tilde{d}}_{wolf}$)	-0.05	**-**	[-0.09; -0.01]	-
log (*d*_*wolf*_)	**-**	0.03	**-**	[0.0; 0.06]
*Interaction (Wolf UD*, log (*d*_*wolf*_)*)*	**-**	-0.03	**-**	[-0.06; 0.0]
**Random (variance)**				
ID	0.18	**-**	**-**	**-**

*reference category is *day*

## Discussion

We extended the HMM-SSF in a way that it can now be used, with large datasets that include missing data and irregular time intervals, to assess population-level habitat selection during sequential behavioral bouts along animal trajectories, in different ecological contexts. Our simulation analysis illustrated the robustness of the HMM-SSF to adequately characterize factors creating movement biases during alternative movement modes under various ecological contexts. We also demonstrated that the HMM-SSF provided more realistic habitat selection estimates than a 2-step approach. Further, we used empirical movement data to illustrate the versatility of the HMM-SSF. Accordingly, the HMM-SSF could become a prevailing tool to assess how animal species use their environment in relation to the complex interplay between their need to move, their knowledge of the environment and navigation capacity, their motion capacity, and the external factors related to landscape heterogeneity.

To date, the tools that have been developed to identify behavioral bouts are, for the most part, applied to movement data from a single individual and without considering habitat selection [8, 21, 42] [but see also 30], thus limiting the scope of potential conclusions. Here, we first fit a HMM-SSF to each individual’s trajectory, then we combined them using an appropriately weighted average (see Eq 4) of the HMM-SSF of all individuals to infer habitat selection at the population level. To do so, we took advantage of the fact that the maximum likelihood estimators of the selection coefficients in each individual HMM-SSF have a distribution that is approximately normal with variance matrix that is easily estimated [27, 43]. Some studies have assessed habitat selection for each behavioral mode by proceeding in a second analysis [23, 25], however habitat covariates are not included to identify movement modes making them less biologically relevant. Additionally, parameters of the selection model are also less accurately estimated with this approach, due to an underestimation of the error in selection coefficient estimates. We clearly demonstrated that the HMM-SSF performed better to assess behavior-specific habitat use from large datasets of movement data including multiple individuals than a 2-step approach. The HMM-SSF has large scope since strong predictive power on animal space use fundamentally relies on gaining a mechanistic understanding of animal movement modes [44, 45]. Specifically, the HMM-SSF provides a tool to identify movement modes together with their underlying cause and interplay with environmental attributes, in different contexts. For example, the HMM-SSF revealed that zebras selected locations closer to waterholes during inter-patch movements (i.e., in travelling mode) whereas they selected locations further from waterholes during intra-patch movements (i.e., in encamped mode, Table 3, Fig 4 and S3 Fig). Thus, these contrasted selection patterns could not be perceived when habitat selection was assessed without considering different phases of animal behavior (one state SSF suggested selection for areas closer to water, not shown).

In addition to the improved accuracy of the estimates provided by the HMM-SSF, our extensions also contribute to a more practical framework. Particularly, we developed the HMM-SSF such that it is now able to deal with data that are missing or collected at irregular time intervals. In such case, an individual trajectory is made of several successive segments separated by gaps or missing data. We assumed and treated those segments as independent to each other and multiplied their likelihood to estimate model parameters for an individual trajectory. A violation of this assumption could lead to biased estimates of variance of selection coefficients, although our evaluation of model performance using *k*-fold cross validation revealed very high predictive capacity for all models (Table 3). Such extension is a crucial asset of the HMM-SSF since it avoids the preliminary step of data regularizing often compulsory due to gaps and irregular time intervals commonly observed in movement datasets obtained from telemetry [42, 46]. Besides, the HMM-SSF provides a framework that only requires drawing random distances and random angles to sample random steps, and extract landscape attributes associated to observed and random steps which makes it easy-to-use with relatively low computational cost. Indeed, the HMM-SSF took between several hours to several days (<1 week) to fit the different datasets. Finally, the HMM-SSF is not restricted by the number of parameters, which can be computationally demanding in other types of state-space models [8, 31, 47].

Simulation analysis illustrated the robustness of the HMM-SSF even though the assumptions of the HMM-SSF are violated. For example, in the simulations based on state-specific step selection functions, we simulated transition rules based on environmental covariates whereas the HMM-SSF supposes that transition are governed by a Markov chain [27]. Because this assumption is likely not biologically reasonable [5], misspecification could potentially lead to false inference [18]. For example, Gurarie et al. [18] revealed that, out of four tools tested, the first passage time analysis was the only one to perform well at correctly partitioning a simulated trajectory into movement modes, when the behavioral switch was defined in terms of spatial attributes. However, the first passage time provided only distinct movement modes but no habitat selection analysis [3, 18]. From our analysis of the simulated data, the HMM-SSF showed high predictive power in both the simulation studies and empirical applications (Tables 1–3). The HMM-SSF proceeded iteratively to both identify the movement modes along individual trajectories and estimate selection coefficient for each mode, thereby increasing the power of the method to characterize the hidden states [27]. Thus, the HMM-SSF provided valid inferences on the underlying selection processes associated to movement modes from different behavioral processes.

We also illustrated the versatility of the HMM-SSF by assessing behavior-specific habitat selection along individual trajectories in three different ecological contexts. Multi-state analysis applied to individual trajectory has classically been used to assess the spatio-temporal scale over which foraging process occurs [3, 48]. Here, we illustrate how the HMM-SSF can be helpful to assist in the identification of the onset of migration and evaluate how movement is dictated by habitat features during the different phases of the migratory behavior [49]. Considering the different phases of the migratory behavior into habitat selection analysis of migratory movement can increase our mechanistic understanding of animal space use during migration [10]. Mule deer use stopovers as stepping stones during their migration, from which they derive foraging benefits [49, 50]. Whereas Merkle et al. [35] showed that aspect generally influenced mule deer movements during migration, the HMM-SSF revealed that deer selected southerly aspect locations solely during stopovers and not during travelling mode (Table 3). This makes sense as mule deer spend 95% of their time on stopovers foraging on early green-up occurring on southerly aspects in spring [50]. Mule deer also oriented their migratory movement towards their previously visited summer range (Fig 3), a typical behavior among migrants [51, 52]. Not only does our approach provide results consistent with previous studies, but it does so by providing statistical models of 2-state migratory behaviors that accounted for the entire dataset in a single analysis. The approach further provides the relative probability, over space and time, that deer were in traveling mode instead of at a stopover.

In addition, we evaluated behavior-habitat specific relationship during movement modes of resident animals and assessed the circumstances over which a forager avoids a nearby predator. The model showed that bison response to the presence of wolves was strongly context dependent. Bison appeared especially apprehensive when they were in areas used intensively by wolves, as they reacted more promptly to the nearby presence of a wolf by being more likely to switch to travel mode. Similarly, zebras are far more likely to flee immediately than stay when an encounter with lion (*Panthera leo*) occurs in bushy areas, where zebras are more easily ambushed [38]. As the spatial and temporal dynamics of reactive prey response to predator encounter remains poorly known [38], we illustrated how the HMM-SSF can help identifying the spatial context over which prey respond to predator presence. The HMM-SSF could potentially be used to assess additional spatial processes that involve successive behavioral bouts (e.g., dispersal behavior, central place foraging, [53]). Besides, the HMM-SSF can also accommodate more than two movement modes when the temporal frequency of GPS relocations is high enough to distinguish between the resting and the foraging periods [7, 54].

In conclusion, HMM-SSF provides a robust, flexible and relatively straightforward tool to gain a mechanistic understanding of animal movement in different ecological contexts and properly assess which strategies animals use to exploit a heterogeneous environment. Specifically, our model identifies behavioral phases along animal trajectories, together with the interplay between movement modes and environmental attributes. The HMM-SSF can also be used to determine the factors influencing transitions between movement modes, thus giving an opportunity to identify the underlying mechanisms of the temporal dynamics of animal movement.

## Supporting information

S1 Appendix Calculation of average travelled distance using coefficient estimates associated to step length. (PDF)

S1 Table Values and definition [from c] of model parameters used to simulate multi-state correlated random walks in three scenarios of landscape patchiness. (PDF)

S2 TableCoefficient estimates along with their 95% confidence interval (95% CI) of the mixed-effects generalized linear model with binomial distribution (HMM-SSF + GLMM) and the multi-state correlated random walk model (HMM-CRW) to predict probability of switching from encamped to travelling mode, in 500 simulated foragers moving among resource patches and avoiding a predator.*In resource patch* is a dummy variable indicating whether the forager is within a resource patch (i.e., patch quality >0), ${\tilde{d}}_{Predator}$ equals the actual distance of the predator from the forager (*d*_*Predator*_) when *d*_*Predator*_ ≤ 0.8 km and 0.8 km, otherwise. *log*(*d*_*Predator*_) is the natural logarithm of *d*_*Predator*_.(PDF)

S3 TableCoefficient estimates along with their 95% confidence interval (95% CI) of mixed-effects generalized linear models with binomial distribution to predict probability of switching from encamped to travelling mode of movement, in plains bison during summer in Prince Albert National Park (SK, Canada).Each table represents estimates for a specific threshold probability (*Pthreshold*) used to categorized transition and non-transition from the conditional probabilities of being in encamped or travelling state, obtained from the fit of the HMM-SSF to plains bison trajectories. ${\tilde{d}}_{wolf}$ was set to the actual distance between bison and wolf (*d*_*wolf*_) when *d*_*wolf*_≤*d*_*threshold*_ and *d*_*threshold*_, otherwise.(PDF)

S1 Fig Simulated heterogeneous landscape used in the multi-state biased correlated random walk simulations, from gaussian random field with an exponential covariance function with variance = 1, nugget = 0 and a set of patch concentration (*μ*_*Q*_) and patch size (*γ*_*Q*_) resulting in three level of patchiness: 1) low (*μ*_*Q*_ = -1.5, *γ*_*Q*_ = 2), 2) intermediate (*μ*_*Q*_ = -0.5, *γ*_*Q*_ = 2) and 3) high (*μ*_*Q*_ = 1, *γ*_*Q*_ = 10). (PDF)

S2 Fig Distribution of distance to the closest waterhole according to the mode of movement estimated from the HMM-SSF for 18 zebras in Hwange National Park during the dry hot season. The conditional probabilities of being in each state, obtained from the fit of the HMM-SFF, were dichotomized to 0–1 based on a 0.5 threshold to determine the state of the individual at each step on its trajectory.
(PDF)

S3 Fig Log-likelihood profile from mixed-effects generalized linear model with binomial distribution to predict probability of switching from encamped to travelling mode of movement, according to a gradient of threshold distance, *d*_*threshold*_. (PDF)

S4 Fig Total number of switches from encamped to travelling mode of movement according to day time, estimated using conditional probabilities of being in each state, obtained from the fit of the HMM-SFF to plains bison trajectories followed during the summers 2005–2016. We then separated the day in four periods: Night: 22:00–02:00, Dawn: 03:00–06:00, Day: 07:00–15:00 and Dusk: 16:00–21:00.
(PDF)
