# Supplementary material for: Multi-mode movement decisions across widely ranging behavioral processes
Source: PLoS One. 2022 Aug 11;17(8):e0272538. doi: 10.1371/journal.pone.0272538 (PMC9371300; doi:10.1371/journal.pone.0272538)
Supplement: S1 Appendix — (PDF) [file pone.0272538.s001.pdf]

Supplementary Information Text

S1 Appendix. Calculation of average travelled distance using coefficient estimates associated to step length.

In our statistical model, we suppose that step length distribution takes the following form:

$$f(d) \propto d^{\beta_{log.step\ legnth}} e^{\beta_{step\ length} d}, d > 0 \qquad [1]$$

where, d is the step length, $\beta_{log.step\ legnth}$ and $\beta_{step\ length}$ are the selection coefficients associated to log (d) and d, respectively [1]. If $\beta_{log.step\ legnth}$> -1 and $\beta_{step\ length}$< 0, then Eq. 1 corresponds to a gamma distribution for step length, such that the average step length can be calculated using:

$$\bar{d} \approx \frac{\beta_{log.step\ legnth} + 1}{-\beta_{step\ length}} \qquad [2]$$

However, when $\beta_{log.step\ legnth}$< -1 or $\beta_{step\ length}$> 0, Eq. 1 does not correspond to a gamma distribution such that we used Metropolis algorithm to simulate 20,000 step lengths from Eq. 1, and calculate the average step length from the last 10,000 [2].

References

1. Nicosia A, Duchesne T, Rivest L-P, Fortin D (2017) A multi-state conditional logistic regression model for the analysis of animal movement. The Annals of Applied Statistics 11: 1537-1560.
2. Robert CP, Casella G (2004) The Metropolis—Hastings Algorithm. Monte Carlo Statistical Methods: Springer, New York, NY. pp. 267-320.
