# Supplementary material for: Multi-mode movement decisions across widely ranging behavioral processes
Source: PLoS One. 2022 Aug 11;17(8):e0272538. doi: 10.1371/journal.pone.0272538 (PMC9371300; doi:10.1371/journal.pone.0272538)
Supplement: S1 Table — (PDF) [file pone.0272538.s002.pdf]

S1 Table. Values and definition [from [c]] of model parameters used to simulate multi-state correlated random walks in three scenarios of landscape patchiness.

Parameter	Definition	Scenario-specific values		
		Low patchiness	Intermediate patchiness	High patchiness
**Simulations**				
Δt	Model time step	1	1	1
T	Simulation length	500	500	500
**Landscapes**				
μ_Q	Patch concentration	1	-0.5	-1.5
γ_Q	Patch size	10	2	2
**Consumption**				
β_R	Regeneration rate	0.01	0.01	0.01
β_C	Consumption rate	1	1	1
γ_C	Consumption spatial scale	1	1	1
**Memory**[a]				
ψ_M	Short-term memory factor	2	10	5
β_L, β_S	Learning rates	1	1	1
ϕ_L, ϕ_S	Decay rates	0.001, 0.1	0.0001, 0.1	0.0001, 0.1
γ_L, γ_S	Learning spatial scales	1	1	1
**Movement**[b]				
τ_S, τ_F	Autocorrelation time scales	4, 2	4, 2	4, 2
v_S, v_F	Speeds	6, 1	6, 1	6, 1
γ_Z	Memory spatial scale	10	5	10
λ	Mean time to update θ [see [c]]	1	1	1

[a]L = long-term memory, S = short-term memory
[b]S = searching, F = feeding
[c]Bracis C, Gurarie E, Van Moorter B, Goodwin RA (2015) Memory effects on movement behavior in animal foraging. Plos One 10: e0136057.
