# Supplementary material for: Multi-mode movement decisions across widely ranging behavioral processes
Source: PLoS One. 2022 Aug 11;17(8):e0272538. doi: 10.1371/journal.pone.0272538 (PMC9371300; doi:10.1371/journal.pone.0272538)
Supplement: S2 Table — In resource patch is a dummy variable indicating whether the forager is within a resource patch (i.e., patch quality >0), d˜Predator equals the actual distance of the predator from the forager (dPredator) when dPredator ≤ 0.8 km and 0.8 km, otherwise. log(dPredator) is the natural logarithm of dPredator. (PDF) [file pone.0272538.s003.pdf]

S2 Table. Coefficient estimates along with their 95% confidence interval (95% CI) of the mixed-effects generalized linear model with binomial distribution (HMM-SSF + GLMM) and the multi-state correlated random walk model (HMM-CRW) to predict probability of switching from encamped to travelling mode, in 500 simulated foragers moving among resource patches and avoiding a predator. *In resource patch* is a dummy variable indicating whether the forager is within a resource patch (i.e., patch quality >0), $\tilde{d}_{Predator}$ equals the actual distance of the predator from the forager ($d_{Predator}$) when $d_{Predator} \leq 0.8$ km and 0.8 km, otherwise. $log(d_{Predator})$ is the natural logarithm of $d_{Predator}$.

**Effect**	**Estimate**		**95% CI**	
	HMM-SSF + GLMM	HMM-CRW	HMM-SSF + GLMM	HMM-CRW
**Fixed**				
Intercept	3.86	2.11	[3.32 ; 4.40]	[1.99 ; 2.23]
In resource patch (i.e., patch quality > 0)	-1.21	-0.14	[-1.32 ; -1.10]	[-0.18 ; -0.10]
$\tilde{d}_{Predator}$	-8.05	-	[-8.72 ; -7.39]	-
$log(d_{Predator})$	-	-4.87	-	[-4.95 ; -4.80]
**Random (variance)**				
ID	0.09	-	-	-
