# Supplementary material for: Multi-mode movement decisions across widely ranging behavioral processes
Source: PLoS One. 2022 Aug 11;17(8):e0272538. doi: 10.1371/journal.pone.0272538 (PMC9371300; doi:10.1371/journal.pone.0272538)
Supplement: S3 Table — Each table represents estimates for a specific threshold probability (Pthreshold) used to categorized transition and non-transition from the conditional probabilities of being in encamped or travelling state, obtained from the fit of the HMM-SSF to plains bison trajectories. d˜wolf was set to the actual distance between bison and wolf (dwolf) when dwolf≤dthreshold and dthreshold, otherwise. (PDF) [file pone.0272538.s004.pdf]

S3 Table. Coefficient estimates along with their 95% confidence interval (95% CI) of mixed-effects generalized linear models with binomial distribution to predict probability of switching from encamped to travelling mode of movement, in plains bison during summer in Prince Albert National Park (SK, Canada). Each table represents estimates for a specific threshold probability ($Pthreshold$) used to categorized transition and non-transition from the conditional probabilities of being in encamped or travelling state, obtained from the fit of the HMM-SSF to plains bison trajectories. $\tilde{d}_{wolf}$ was set to the actual distance between bison and wolf (d_{wolf}) when $d_{wolf} \leq d_{threshold}$ and $d_{threshold}$, otherwise.

A. $\boldsymbol{Pthreshold = 0.6}$

Effect	Estimate	95% CI
**Fixed**		
Intercept	-3.26	[- 3.67 ;-2.85]
*Period of day**		
Dawn-Dusk	0.41	[0.28 ; 0.54]
Night	-1.78	[-2.09 ; -1.47]
Meadow	-0.29	[-0.43 ; -0.16]
Wolf UD	0.17	[0.02 ; 0.32]
$\tilde{d}_{wolf}{}^{\dagger}$	0.06	[-0.07 ; 0.18]
Interaction (Wolf UD, $\tilde{d}_{wolf}{}^{\dagger}$)	-0.06	[-0.11 ; 0]
**Random (variance)**		
ID	0.16	

† $d_{threshold}$ = 3.2 km

B. $\boldsymbol{Pthreshold = 0.7}$

Effect	Estimate	95% CI
**Fixed**		
Intercept	-3.53	[-4.04 ; -3.0]
*Period of day**		
Dawn-Dusk	0.41	[0.24 ; 0.58]
Night	-1.93	[-2.35 ; -1.51]
Meadow	-0.38	[-0.56 ; -0.20]
Wolf UD	0.20	[0.01 ; 0.38]

$\tilde{d}_{wolf}{}^{\dagger}$	-0.01	[-0.17 ; 0.19]
Interaction (Wolf UD, $\tilde{d}_{wolf}{}^{\dagger}$*)*	-0.09	[-0.16 ; -0.01]
**Random (variance)**		
ID	0.12	

† $d_{threshold} = 2.9$ km

C. $\boldsymbol{Pthreshold = 0.8}$

Effect	Estimate	95% CI
**Fixed**		
Intercept	-2.23	[-3.46 ; -1.0]
*Period of day**		
Dawn-Dusk	0.36	[0.13 ; 0.58]
Night	-2.25	[-2.89 ; -1.61]
Meadow	-0.54	[-0.79 ; -0.29]
Wolf UD	-0.29	[-0.89 ; 0.31]
$\tilde{d}_{wolf}{}^{\dagger}$	-1.73	[-2.96 ; -0.50]
Interaction (Wolf UD, $\tilde{d}_{wolf}{}^{\dagger}$*)*	0.28	[-0.34 ; 0.91]
**Random (variance)**		
ID	0.06	

*reference category is *day*

† $d_{threshold} = 1.0$ km
