# Supplementary material for: Multi-mode movement decisions across widely ranging behavioral processes
Source: PLoS One. 2022 Aug 11;17(8):e0272538. doi: 10.1371/journal.pone.0272538 (PMC9371300; doi:10.1371/journal.pone.0272538)
Supplement: S1 Fig — (PDF) [file pone.0272538.s005.pdf]

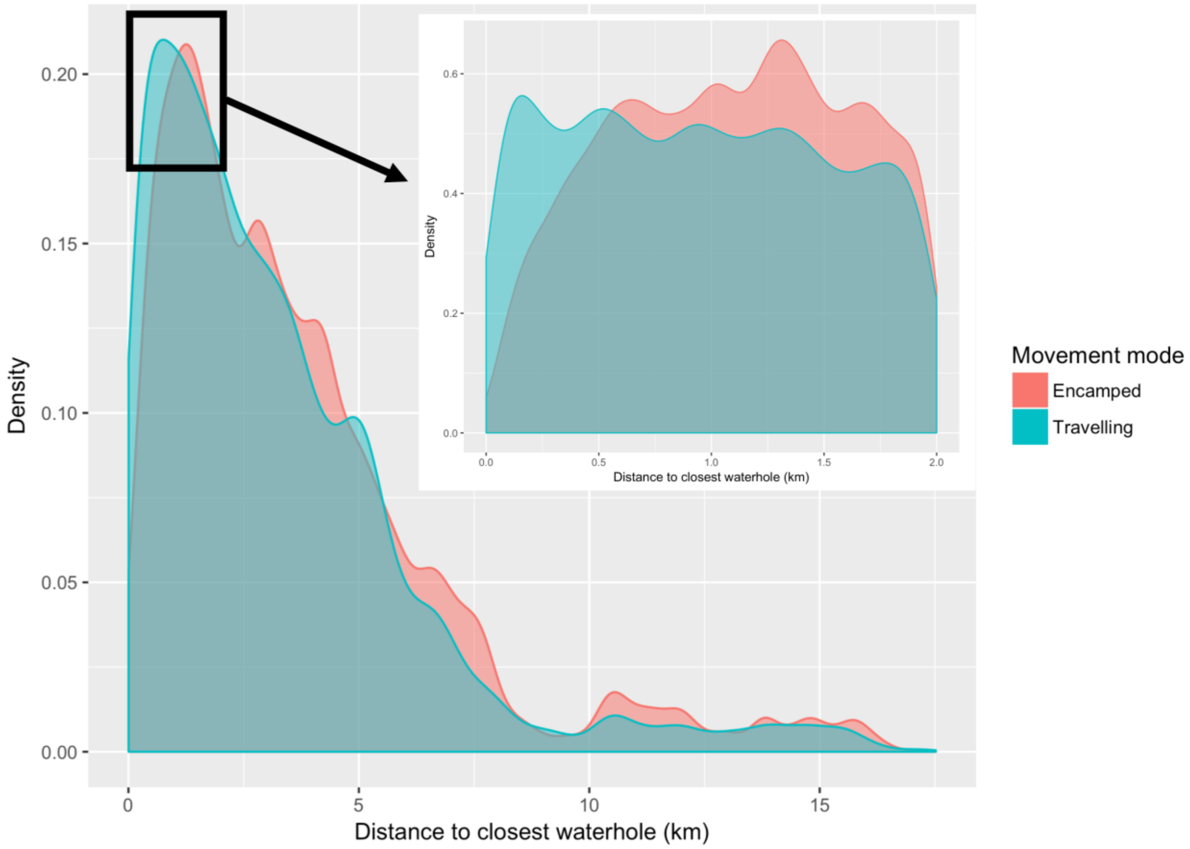

S1 Fig. Distribution of distance to the closest waterhole according to the mode of movement estimated from the HMM-SSF for 18 zebras in Hwange National Park during the dry hot season. The conditional probabilities of being in each state, obtained from the fit of the HMM-SFF, were dichotomized to 0-1 based on a 0.5 threshold to determine the state of the individual at each step on its trajectory.
