# Supplementary material for: Multi-mode movement decisions across widely ranging behavioral processes
Source: PLoS One. 2022 Aug 11;17(8):e0272538. doi: 10.1371/journal.pone.0272538 (PMC9371300; doi:10.1371/journal.pone.0272538)
Supplement: S2 Fig — The conditional probabilities of being in each state, obtained from the fit of the HMM-SFF, were dichotomized to 0–1 based on a 0.5 threshold to determine the state of the individual at each step on its trajectory. (PDF) [file pone.0272538.s006.pdf]

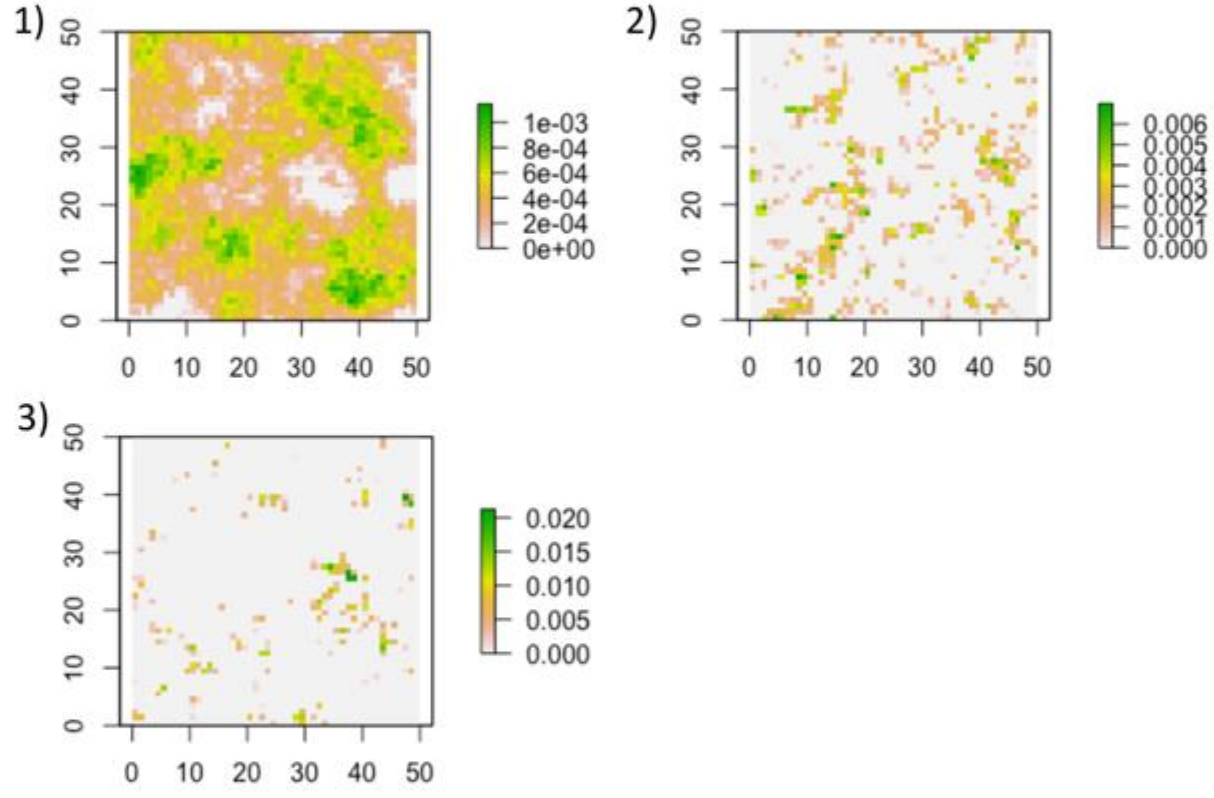

S2 Fig. Simulated heterogeneous landscape used in the multi-state biased correlated random walk simulations, from Gaussian random field with an exponential covariance function with variance = 1, nugget = 0 and a set of patch concentration (μ_Q) and patch size (γ_Q) resulting in three level of patchiness: 1) low (μ_Q = -1.5, γ_Q = 2), 2) intermediate (μ_Q = -0.5, γ_Q = 2) and 3) high (μ_Q = 1, γ_Q = 10).
