# Supplementary material for: Multi-mode movement decisions across widely ranging behavioral processes
Source: PLoS One. 2022 Aug 11;17(8):e0272538. doi: 10.1371/journal.pone.0272538 (PMC9371300; doi:10.1371/journal.pone.0272538)
Supplement: S3 Fig — (PDF) [file pone.0272538.s007.pdf]

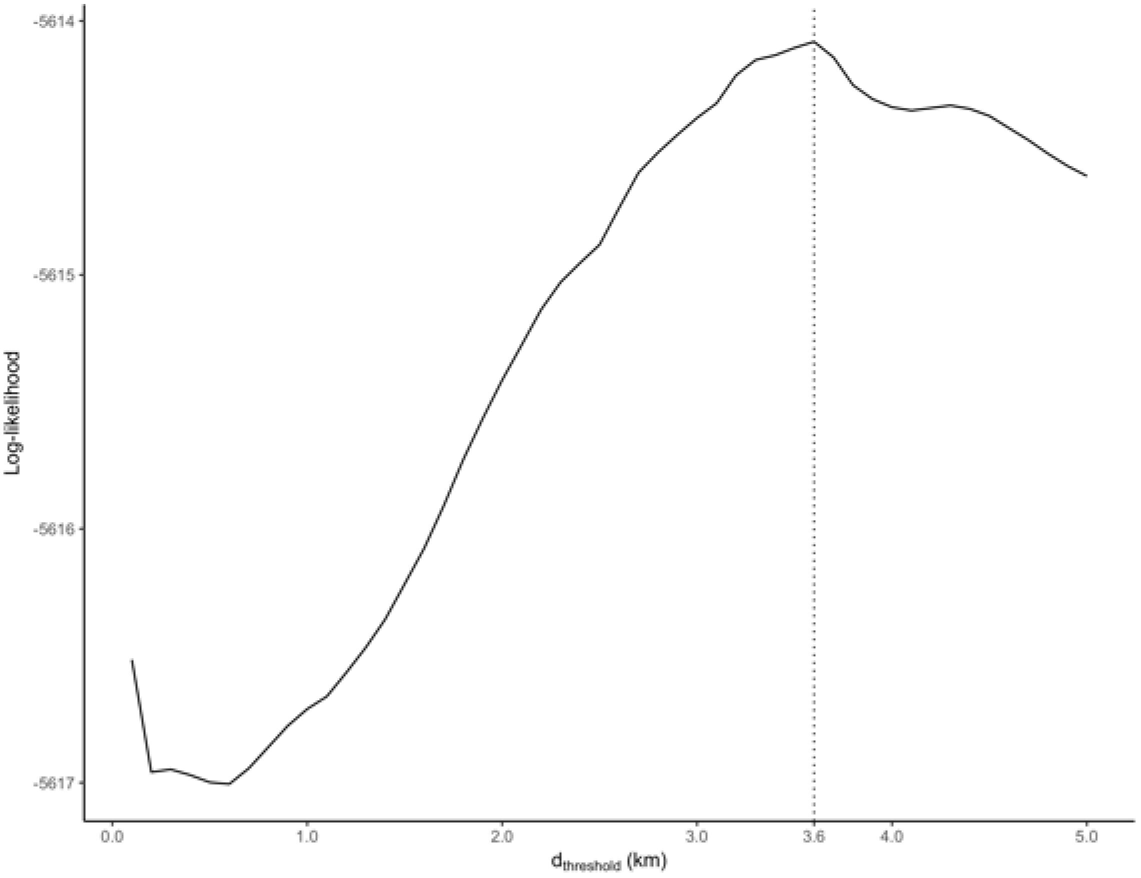

S3 Fig. Log-Likelihood profile from mixed-effects generalized linear model with binomial distribution to predict probability of switching from encamped to travelling mode of movement, according to a gradient of threshold distance, $d_{threshold}$.
