# Supplementary material for: Multi-mode movement decisions across widely ranging behavioral processes
Source: PLoS One. 2022 Aug 11;17(8):e0272538. doi: 10.1371/journal.pone.0272538 (PMC9371300; doi:10.1371/journal.pone.0272538)
Supplement: S4 Fig — We then separated the day in four periods: Night: 22:00–02:00, Dawn: 03:00–06:00, Day: 07:00–15:00 and Dusk: 16:00–21:00. (PDF) [file pone.0272538.s008.pdf]

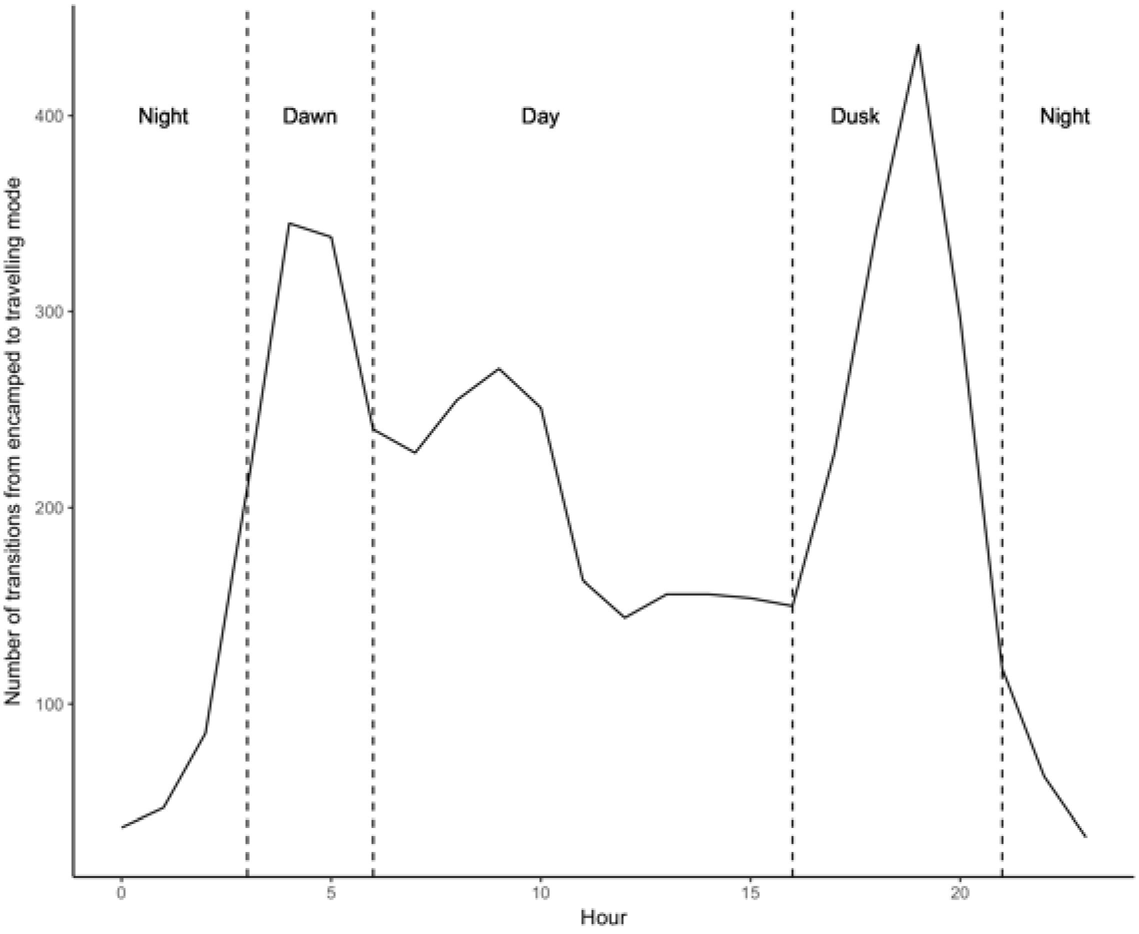

S4 Fig. Total number of switches from encamped to travelling mode of movement according to day time, estimated using conditional probabilities of being in each state, obtained from the fit of the HMM-SFF to plains bison trajectories followed during the summers 2005-2016. We then separated the day in four periods: Night: 22:00-02:00, Dawn: 03:00-06:00, Day: 07:00-15:00 and Dusk: 16:00-21:00.
